# Biodiversity of the Deep-Sea Continental Margin Bordering the Gulf of Maine (NW Atlantic): Relationships among Sub-Regions and to Shelf Systems

**DOI:** 10.1371/journal.pone.0013832

**Published:** 2010-11-19

**Authors:** Noreen E. Kelly, Elizabeth K. Shea, Anna Metaxas, Richard L. Haedrich, Peter J. Auster

**Affiliations:** 1 Centre for Marine Biodiversity, Bedford Institute of Oceanography, Dartmouth, Nova Scotia, Canada; 2 Department of Mollusks, Delaware Museum of Natural History, Wilmington, Delaware, United States of America; 3 Department of Oceanography, Dalhousie University, Halifax, Canada; 4 Department of Biology, Memorial University, St. John's, Canada; 5 Department of Marine Sciences and Northeast Underwater Research Technology & Education Center, University of Connecticut, Groton, Connecticut, United States of America; American Museum of Natural History, United States of America

## Abstract

**Background:**

In contrast to the well-studied continental shelf region of the Gulf of Maine, fundamental questions regarding the diversity, distribution, and abundance of species living in deep-sea habitats along the adjacent continental margin remain unanswered. Lack of such knowledge precludes a greater understanding of the Gulf of Maine ecosystem and limits development of alternatives for conservation and management.

**Methodology/Principal Findings:**

We use data from the published literature, unpublished studies, museum records and online sources, to: (1) assess the current state of knowledge of species diversity in the deep-sea habitats adjacent to the Gulf of Maine (39–43°N, 63–71°W, 150–3000 m depth); (2) compare patterns of taxonomic diversity and distribution of megafaunal and macrofaunal species among six distinct sub-regions and to the continental shelf; and (3) estimate the amount of unknown diversity in the region. Known diversity for the deep-sea region is 1,671 species; most are narrowly distributed and known to occur within only one sub-region. The number of species varies by sub-region and is directly related to sampling effort occurring within each. Fishes, corals, decapod crustaceans, molluscs, and echinoderms are relatively well known, while most other taxonomic groups are poorly known. Taxonomic diversity decreases with increasing distance from the continental shelf and with changes in benthic topography. Low similarity in faunal composition suggests the deep-sea region harbours faunal communities distinct from those of the continental shelf. Non-parametric estimators of species richness suggest a minimum of 50% of the deep-sea species inventory remains to be discovered.

**Conclusions/Significance:**

The current state of knowledge of biodiversity in this deep-sea region is rudimentary. Our ability to answer questions is hampered by a lack of sufficient data for many taxonomic groups, which is constrained by sampling biases, life-history characteristics of target species, and the lack of trained taxonomists.

## Introduction

The deep sea, defined here as depths below the shelf break at ∼200 m [1], is the largest ecosystem on the planet. It comprises approximately 63% of the surface of the Earth and provides a number of vital ecosystem functions, including nutrient recycling, carbon sequestration, and regulation of ocean chemistry [2]. The deep sea contains a multitude of distinct habitats, from the rocky mid-ocean ridges, submarine canyons, trenches and seamounts, to the island-like chemoautotrophic cold seeps, hydrothermal vents and whale-falls, and sediment-dominated continental slopes and abyssal plains [1]. With the exception of chemoautotrophic communities, organisms in the deep sea are typically food-limited, and rely on sinking organic material produced in surface waters [3]. In addition to low biological productivity, most deep seafloor habitats are typically characterized by low physical energy, low temperatures (−1–4°C), an absence of sunlight, and low sediment accumulation rates, supporting communities with low biomass, growth rate, rate of reproduction and recruitment [1], [4]. However, canyons and seamounts foster higher biomass communities by enhancing bottom currents (and thus supply of food particles) and/or flux of organic matter [4]–[6]. Despite these conditions, it is clear that deep-sea habitats harbour high species diversity; estimates of richness frequently exceed 1 million species globally [7]–[10]. However, deep-sea research has lagged far behind that in other habitats, and many basic questions remain to be addressed [2].

The Census of Marine Life (CoML) program (http://www.coml.org/) is a global network of marine scientists engaged in a 10-year mission to assess and explain the diversity, distribution, and abundance of life in the oceans. As part of CoML, the Gulf of Maine Area (GoMA) program (http://www.gulfofmaine-census.org/) was designed to advance knowledge of biodiversity patterns and ecological processes over a range of habitats and taxonomic groups in the Gulf of Maine, and develop an ecosystem-scale understanding of biodiversity as a foundation for an ecosystem-approach to conservation and management. While the shelf region of the Gulf of Maine is relatively well-studied, fundamental questions regarding the diversity, distribution, and abundance of species living in deep-sea habitats along the continental margin, and how they are linked to shelf communities and processes, are currently unanswered. Lack of such knowledge precludes a more full understanding of the function of the Gulf of Maine ecosystem as a whole. With these goals in mind, we assess here the current state of ecological knowledge about deep-sea species and habitats of the continental margin bordering the Gulf of Maine.

The geological, bathymetric, and hypsometric characteristics of the deep-sea portion of the Gulf of Maine are generally well known [11]. Six distinct sub-regions can be identified within the overall area: the continental shelf-slope break, continental slope, NE Channel, continental rise, submarine canyons, and Bear Seamount (the western-most of the New England Seamount chain). The continental slope spans the outer margin of the continental shelf, beginning at the continental shelf break at 60–200 m and extending to a depth of 2000 m. It has an average gradient of 3–6°, and is dominated by sand above 300 m, but the proportions of silt, clay and mud increase with depth [12], [13]. Below 2000 m, a marked decrease in seafloor gradient with only gradual changes in surficial topography delimits the beginning of the continental rise. The slope relief is moderately homogeneous except where it is cut by submarine canyons [14], [15]. These canyons have steep walls with outcroppings of bedrock and clay, and are continuous from the canyon heads at the continental shelf-slope break down to the base of the continental slope or rise [16]. The Northeast Channel, between Georges Bank and Browns Bank, is comprised of three steep canyons that drop into depths of 1000 m bounded by flat sandy bottoms, although much of the substrate is a mixture of pebble, cobble, boulders and rocky outcrops [17], [18]. Bear Seamount rises out of the continental slope at depths of 2000–3000 m to a generally flat summit at 1100 m depth, and is comprised of outcrops of basaltic rock and scattered glacial erratics of various sizes with some areas composed of a thick sedimentary drape [19].

The oceanography of the continental margin is characterized as a three layer system: (1) a warmer surface layer (>17°C) which penetrates to 200 m and only exists during the summer months due to seasonal warming; (2) a middle layer between 200–600 m encompassing a permanent thermocline (ranging from 4–17°C); and (3) a deep, cold layer (≤4°C) comprising two-thirds of the water column [11], [13]. Below 600 m, temperatures decrease at the rate of ∼0.02°C m^−1^, and average ∼2°C at 4000 m. Warm-core (anti-cyclonic) rings that spin off the Gulf Stream are the principal source of variability in slope waters, and introduce water with different properties into the major slope water masses in the upper 1000 m [20]. Currents along the continental slope are isolated from the Gulf of Maine by Georges Bank, the eastern coastal shelf and the Scotian shelf, but also affect the most western of the New England Seamounts. The Northeast Channel is one of the primary avenues for exchange of water between the Gulf of Maine and the North Atlantic Ocean, as warmer more saline slope water enters at the northeastern edge of the channel, while colder surface water exits at the southwestern edge [21], [22]. Currents are typically rectified by topography, and tend to intensify within canyons, leading to enhanced mixing and sediment transport in the area [23]. Bear Seamount is influenced by the Gulf Stream and associated eddies, the Deep Western Boundary Current and Antarctic Bottom Water at the base, and thus experiences colder water conditions than the surrounding deep-water habitats [19], [24]. The variations in surficial geology and topography, substratum properties, sediment grain size composition, hydrodynamic regime, temperature, water masses properties, and depth range among these sub-regions undoubtedly influence the diversity and structure of faunal communities inhabiting these distinct habitats.

Previous studies investigating deep-sea communities in the Gulf of Maine region have focussed on a specific habitat (e.g. continental slope [25], [26]; canyons [27]–[29]; Bear Seamount [19], [30]) or a specific taxon or faunal type (e.g. corals [18], [31]–[34]; macrofauna [35], [36]; fishes [37], [38]), limiting the ability to estimate diversity across regional scales or broad taxonomic groups. However, these studies have marvelled at the unexpected diversity and abundance found within specific habitats, and documented the discovery of species new to science or previously unknown in the region. Together these studies paint a picture of a deep-sea region comprised of distinct and diverse faunal communities. Currently, the number of different species inhabiting the deep-sea Gulf of Maine region, their distribution across habitats, or their connection to faunal communities of the continental shelf is poorly understood. Given the number of recent and on-going scientific exploratory and sampling-intensive expeditions into this area, and the impending threat of increased exploitation of deep-water fisheries and natural oil and gas extraction (e.g. [39]–[41]), a comprehensive synthesis of species distributions and patterns of biodiversity would provide a baseline of biodiversity in this region and shed more light onto the importance of deep-sea species to the Gulf of Maine. Such a synthesis is needed in order to move forward with both scientific and management activities in the region.

Limitations in sampling extent and effort, as well as accessibility of data, across the census area present several challenges to compiling a synthesis of biodiversity in the deep-sea Gulf of Maine region. Sampling efforts are still mostly exploratory (e.g. trawling around seamounts; [19], [30]; submersible video surveys in the NE Channel and the continental slope, Metaxas unpubl.), and the lack of regular standardized sampling in space and time prevents the categorization of most faunal groups across habitats. The deep-sea Gulf of Maine region covers a large spatial area (>25 000 km^2^; [11]). A lack of widespread sampling for characterization and no on-going long term monitoring in this region prevents quantification of temporal patterns in diversity or abundance, as well as the detection of changes in these metrics by natural or anthropogenic disturbances. Methods of sampling differ by target species or taxonomic group, as well as substrate type, yielding different estimates of diversity or abundance for different taxonomic groups within and among habitats (e.g. [28], [42]).

In this paper, we synthesize the current state of knowledge of species diversity in the deep-sea areas adjacent to the continental shelf of the Gulf of Maine by providing an original analysis of patterns of diversity and species distribution through the compilation of data from a variety of published and unpublished studies, museum and government records, and online sources. Given the limitations in sampling extent and effort and accessibility of data previously mentioned, here we focus primarily on the broad-scale patterns of diversity, distribution, and abundance of megafaunal (>∼5 cm) and macrofaunal (>0.5 mm) species. We identify taxonomic gaps in our knowledge and provide estimates of the potential total diversity in the deep-sea region. We review previous studies to discuss the environmental and ecological drivers of diversity for relatively well-studied, distinct habitats within the deep-sea portion of the Gulf of Maine. Finally, we provide our collective perspectives on the most pressing questions, research needs, and technology issues required to increase our understanding of deep-sea diversity in this region. This paper is a synthesis product of the GoMA project of the CoML program, and forms part of a broader overview of the biodiversity of marine life in the Gulf of Maine area.

## Methods

### Study area

**Figure 1:**
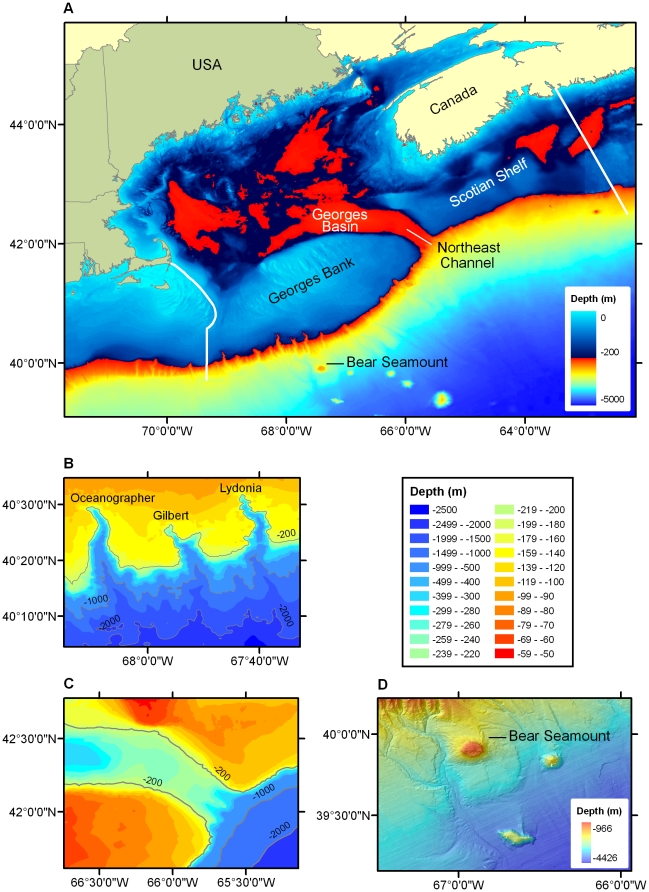
The deep-sea Gulf of Maine census area. A) The deep-sea Gulf of Maine census area, showing eastern and western boundaries of Gulf of Maine Area Program in white. Note use of two depth scales in the color bar. B) Canyons, continental slope and shelf edge of Georges Bank NW of Bear Seamount. Depth scale is shown on the right-hand side. C) NE Channel and slope. Depth scale is same as in B. D) Bear Seamount (summit depth ∼1100 m). Other seamounts shown are Physalia (to the east) and Mytilus (southeast). Depths in panels A, B and C are from the USGS Digital Bathymetry for the Gulf of Maine (∼500m/pixel). Panel A was augmented with data from the General Bathymetric Chart of the Oceans [GEBCO (∼1000m/pixel)]. Depths in panel D are from high resolution (100m/pixel) bathymetric data processed by the Center for Coastal and Ocean Mapping/Joint Hydrographic Center, University of New Hampshire.

We defined the deep-sea region of the Gulf of Maine based on a combination of latitude, longitude, and depth. For our purposes, the region is contained by 39–43°N, 63–71°W, from 150–3000 m depth (Fig. 1). To examine patterns of biodiversity within the area, we further divided this region into 6 broad, yet distinct habitat types, which we term ‘sub-regions’. These sub-regions were defined based on differences in bathymetry and topographic relief, changes in substrate composition and current regimes, and potential linkages to the continental shelf of the Gulf of Maine or the deep North Atlantic (Table 1). These sub-regions comprise not only benthic habitats, but also the mesopelagic and bathypelagic zones of the water column, extending from a depth of 150 m to the ocean floor.

**Table 1:** Definitions of sub-regions used in this study.

Sub-region	Depth	Gradient/Relief	Sediment composition	Linkages	Additional characteristics
Shelf Edge	150–300 m	7–8°	Primarily sandy	Shelf/slope boundary	Upper portion of the continental shelf; location of the shelf-slope front water mass
Continental Slope	300–2000 m	2–12°, but typically 6°	Silts and clays, but occasional boulders and pockets of sand present	Transition between the continental shelf and the deeper waters of the North Atlantic	
Canyons	Transect the Continental Slope; largest are 400 m deep and 5 km wide	Highly variable, ranging from 3–70°	Highly variable, ranging from mud/slit/clays to boulder fields and rocky outcroppings	Areas of strong currents rectified by bottom topography and areas of sediment transport from the continental shelf Gulf of Maine to the deep Atlantic	Majority are located along the southern edge of Georges Bank
Seamount (Bear)	Summit occurs at 1100 m below sea level and extends to 3000 m on the seaward edge	Steep sides that descend over 1000 m to the seafloor	Volcanic rock substrate, but partially buried by the deposition of fine sediments	Geographically isolated from the Continental slope; may act as stepping stone for spread of species across the Atlantic	Oldest and western most seamount of the Western New England seamount chain
NE Channel	210–900 m	Deep valley between Georges Bank and Browns Bank	Poorly sorted mixture of pebble, cobble and boulder, with stretches of sand	Main connection between the continental shelf Gulf of Maine and the Continental slope	Major site of water mass exchange
Continental Rise	>2000 m depth	Relatively flat	Fine grained sediments, silts and clays	Transition between slope and abyssal plain/deep Atlantic	

### Patterns of known biodiversity

To estimate the known biodiversity within the deep-sea region of the Gulf of Maine, we created a database of known occurrences of all species based on available data and studies that were conducted within the boundaries of our census area. This database includes records obtained from: (1) the primary (e.g. published in peer-reviewed journals) and secondary (e.g. US and Canadian government technical reports) literature, (2) websites [Ocean Biogeographic Information System (OBIS; www.iobis.org), searched using geographic region], (3) online museum collections [Museum of Comparative Zoology, Harvard University; Peabody Museum of Natural History, Yale University; National Museum of Natural History, Smithsonian Institution], and (4) raw data directly provided by authors (A. Metaxas: NE Channel and Continental Rise macrofauna (sampled as in [18]); P. Auster: canyon head megafauna as in [29] and US coral distributions [32]; R. Haedrich: demersal fish and benthic infauna [43]–[45]). From these sources, we were able to assemble information on benthic, demersal, mesopelagic and bathypelagic taxa, as well as infaunal, macrofaunal, and megafaunal species, comprising exclusively adult life stages. We excluded certain taxonomic groups, namely pelagic zooplankton and upper trophic level predators, such as marine mammals (whales, dolphins, seals) and large predatory or highly migratory species (swordfish, tunas, turtles, jellyfish and siphonophores), due to their transient, anecdotal, or unconfirmed presence within our sub-regions. We also excluded any information on species presence based on gut content analysis of upper trophic level predators sampled within our census area. We used the World Register of Marine Species (WoRMS; www.marinespecies.org) to vet all taxonomic information (spellings, synonyms, validated names). Where our species were not listed on WoRMS (<5% of names), we consulted other sources, namely the Integrated Taxonomic Information System (ITIS; www.itis.gov/index.html), FishBase (www.fishbase.org/search.php), the Encyclopedia of Life (EOL; www.eol.org), and the Global Biodiversity Information Facility data portal (GBIF; http://data.gbif.org). This taxonomic inventory was assembled in Microsoft Access and includes information on geo-referenced occurrences of species (or lowest possible taxonomic resolution) within the census region, any relevant physical (depth, temperature), biological (faunal type, life stage, abundance, condition, microhabitat) or other (date and/or year of sampling, gear type or sampling method) information associated with sampling, and the data source, study, individual, or website from which the information was originally obtained (Data S1, Table S1). This database does not currently include other possible data sources, such as records of species in government databases that are not available to the general public, specimens deposited in museums from recent field studies and not yet identified, or records that are not available electronically.

From the information in this database, we conducted several analyses to characterize and summarize the state of knowledge of biodiversity in this region. It was not possible to adjust for differences in sampling area or effort among the studies and data sources compiled within the database, and thus estimates of abundance and true species richness among sub-regions would have been biased. Instead, we examine broad-scale patterns in the number and distribution of known species, and we conduct an analysis of the taxonomic structure of species lists, within and across sub-regions. For all analyses of known biodiversity, we used only records of occurrences in our database that were identified to the genus or species level.

To examine what is known regarding biodiversity in the study region, we calculated the number of known species and total number of database records within each sub-region and examined the species which dominated our database, determined as the 10 most frequently reported species. To explore how differences in sampling effort among sub-regions have influenced the amount of known biodiversity, we examined the correlation between the total number of database records and the total number of species for each sub-region using a Pearson product-moment correlation for paired samples (one-tailed test, α = 0.025). As a large proportion of the database records contained information on sampling date or year, we used the year of first collection for each species within a sub-region to construct an accumulation curve of species discovery (across all taxonomic groups) over time. A species only contributed to the curve upon first being reported within each sub-region despite any subsequent occurrence(s). Records prior to 1950 were markedly patchy, thus we only included records since this year in the curve. To characterize potential differences in sampling effort among our sub-regions over time, we calculated the cumulative number of records within our database since 1950, as for the species discovery curve, except species records were not excluded on subsequent occurrence(s).

To identify how biodiversity is distributed across taxonomic groups within and among sub-regions, we created tables for taxonomic gap analysis from information in the database. We assigned each species occurrence to a sub-region, based on its associated latitude, longitude, and depth information, or where it was listed as sampled in a specific site within the original study (e.g. Canyons, NE Channel). We then created tables for the number of species occurring within each sub-region grouped by phyla, as well as for taxonomic groups that contained sufficient data (e.g. >10% of the total number of species records) and/or that were also of commercial or scientific interest: (1) Phylum Chordata, Classes Actinopterygii and Elasmobranchii, (2) Phylum Cnidaria, Class Anthozoa, (3) Phylum Arthropoda, Class Malacostraca, (4) Phylum Echinodermata, (5) Phylum Mollusca.

To examine the spatial distribution of biodiversity within and among our sub-regions and identify potential zones of enhanced diversity within our area, we used the geo-referenced occurrence of all species across the study area to create maps for the total number of known species. Where no spatial coordinates were originally provided with a species record, but a specific site was listed in the study (e.g. Oceanographer Canyon, NE Channel), a central latitude and longitude was assigned to those records in order to include them within the appropriate site. Records of species occurrence were imported into ArcGIS 9.2 and intersected with a 0.2 degree grid. Duplicate species records within each grid square were removed, and the unique species remaining in each grid square were then counted and displayed as the total number of known species per grid square. Species divisions were separated into 5 classes, from low to high occurrence, based on the frequency distribution of species. The map was displayed in World Plate Carree. Zones of enhanced diversity within our area were defined as grid squares representing the highest frequency quintile of known species.

The large variation in sampling methods and effort among the studies compiled in our deep-sea database precluded the use of more traditional methods of comparing biodiversity within our system (e.g. comparisons of univariate diversity metrics). Instead, we conducted a taxonomic distinctness analysis [46], [47] to examine whether the species lists from each sub-region have the same biodiversity structure. The taxonomic structure of an assemblage is also an important measure of biodiversity, such that a group of closely related species is regarded as ‘less’ diverse than the same number of more distantly related species [47]. Taxonomic distinctness analysis avoids most problems associated with traditional measures of species richness, and is thus useful for comparing diversity across data sets and studies with uncontrolled, unequal, or unknown degrees of sampling effort, where quantitative data are not available and samples consist of a species list (presence/absence data) [46], [47]. We used two measures of taxonomic relatedness for our species lists (using species within Kingdom Animalia only), which are based on tracing the path through the taxonomic classification tree: (1) average taxonomic distinctness (Δ^+^), the average path length through the taxonomic tree connecting every pair of species in the list, which measures the average degree to which individuals in an assemblage are related to each other [46]; and (2) variation in taxonomic distinctness (Λ^+^), the variance of the taxonomic distance between each pair of species about their mean value Δ^+^, which reflects the unevenness of the taxonomic tree [47]. To create a classification tree for our species lists, we followed the classification provided by WoRMS, and used the Taxon Match Tool to automatically match our species list with their higher classification (e.g. Phylum to Species). Where our species were not listed on WoRMS (<5% of names), we further consulted ITIS, FishBase, EOL, and/or the GBIF data portal, for taxonomic information. We examined whether the taxonomic distinctness measures (Δ^+^, Λ^+^) for the species list for each sub-region fell within the confidence limits generated by 1000 simulations of random subsets of *m* species from the master list (i.e. the total species list from all sub-regions combined) [46]. These randomization procedures test the null hypothesis that a species list from one sub-region has the same taxonomic structure (e.g. diversity) as the master list. All taxonomic distinctness analyses were conducted using PRIMER (Version 6, PRIMER-E Ltd).

We examined potential connections between the deep-sea system and the continental shelf waters (e.g. depth <150 m) of the Gulf of Maine through comparisons of the relatedness in species composition. Also using the PRIMER software package, we calculated a Bray-Curtis similarity coefficient based on presence/absence data to examine the similarity of species lists: (1) among sub-regions, (2) between the entire deep-sea region and the continental shelf, and (3) between deep-sea sub-regions and the continental shelf. The continental shelf species list was compiled from the Gulf of Maine Register of Marine Species (available online: http://research.usm.maine.edu/gulfofmaine-census/about-the-gulf/biodiversity-of-the-gulf/lists), although we excluded specific taxonomic groups to reflect the same taxonomic breadth of the overall deep-sea list (i.e. we excluded all flora, pelagic zooplankton, and upper trophic level predators taxa). In addition, we compared the total number of species in the continental shelf to the entire deep-sea region, to examine the overlap between our deep-sea taxonomic inventory and that of the continental shelf, and to identify the number of species unique to the deep-sea region.

### Patterns of unknown biodiversity

To estimate the potential amount of unknown biodiversity remaining to be discovered in our census region, we constructed species accumulation curves, which plot the increasing total number of different species observed as samples are successively pooled. As this analysis requires the abundance of organisms to be measured using replicated quantitative sampling designs, we were thus restricted to using a subset of studies from within our database which met this criterion. Thus, we report estimates of unknown biodiversity for different sub-regions and different types of organisms which were sampled using different sampling gears (Table 2). To estimate the true total species richness for each sub-region and study, we calculated the non-parametric species richness estimators Chao 1 (abundance-based) and Chao 2 (incidence-based), which attempt to predict the asymptote of the species accumulation curve if the number of samples tends to infinity [48], [49]. To estimate the remaining species richness for sub-regions, we then compared the observed species richness to the estimates provided by the Chao metrics. *EstimateS* (Version 8.0, R. K. Colwell, http://purl.oclc.org/estimates) was used to generate species accumulation curves and species richness estimators (100 randomizations without replacement) for all sub-regions, except for the curves from the Continental Slope, which were calculated in PRIMER, using 999 permutations of sample order and provided by Dr. N. Maciolek (Continental Slope benthic infauna; data from the Atlantic Continental Slope and Rise (ACSAR) North stations 2–8, originally published in [26]).

**Table 2:** Description of studies used to create species accumulation curves for 5 different sub-regions.

Sub-region	Faunal class	Sampling Method	Sampling Year	Depth range (m)	Source
NE Channel	Epibenthic macro- and megafauna	Submersible photographs	2006	450–925	Metaxas, unpublished
Continental Rise	Demersal macrofauna and megafauna	Trawls using 41′ Gulf of Mexico net	1975	2150–2650	[43], [44]
	Epibenthic macro- and megafauna	Submersible photographs	2006	2500–2600	Metaxas, unpublished
Canyon	Epibenthic and demersal megafauna	Submersible photographs	1984	200–350 m	[29]
Continental Slope	Infaunal macrofauna	Sediment cores	1983–1984	550–2180	[26]
Bear Seamount	Mesopelagic and bathypelagic megafauna	Trawls using IGYPT net	2002	362–1475	[30]

## Results

### Patterns of known biodiversity

The deep-sea database for the Gulf of Maine census area currently contains 15 256 records of occurrences across the study region, with 14 320 records resolved to the family level, 13 977 records to the genus level, and 12 249 records to species level. Records were collected between 1874 and 2008, and most are geo-referenced, with associated depth information and method of sampling. However, information on associated physical or chemical variables or the life-history stage of species at time of sampling is absent for most records. The database contains a total of 1671 species for the entire deep-sea region. The Continental Slope currently has the highest number of known species, at 890, while the NE Channel has the lowest, at 136 known species (Table 3). Few species were widespread, as only 90 species were found in ≥4 sub-regions, whereas most were narrowly distributed, with 1093 species occurring in only one sub-region. The 10 most reported species (i.e. those species with the greatest number of occurrence records within the database) within the deep-sea region of the Gulf of Maine were demersal megafaunal species (Table 4).

**Table 3:** Total number of species and number of records of species' occurrence recorded for each sub-region within the deep-sea database.

Sub-region	No. species	No. records
Canyon	326	1299
Continental Rise	227	634
Continental Slope	890	4931
NE Channel	136	1199
Seamount	630	3583
Shelf Edge	314	3573

**Table 4:** Ten most frequently reported species within the deep-sea database.

Phylum	Class	Genus	Species	Common name	No. Records
Chordata	Actinopterygii	*Merluccius*	*bilinearis*	Silver hake	411
Chordata	Actinopterygii	*Urophycis*	*tenuis*	White hake	359
Mollusca	Cephalopoda	*Illex*	*illecebrosus*	Northern shortfin squid	330
Chordata	Actinopterygii	*Phycis*	*chesteri*	Longfin hake	315
Chordata	Actinopterygii	*Helicolenus*	*dactylopterus*	Blackbelly rosefish	310
Chordata	Actinopterygii	*Sebastes*		Redfish	298
Arthropoda	Malacostraca	*Homarus*	*americanus*	American lobster	264
Chordata	Elasmobranchii	*Amblyraja*	*radiata*	Thorny skate	237
Chordata	Actinopterygii	*Glyptocephalus*	*cynoglossus*	Witch flounder	226
Chordata	Actinopterygii	*Urophycis*	*chuss*	Red hake	214

**Figure 2:**
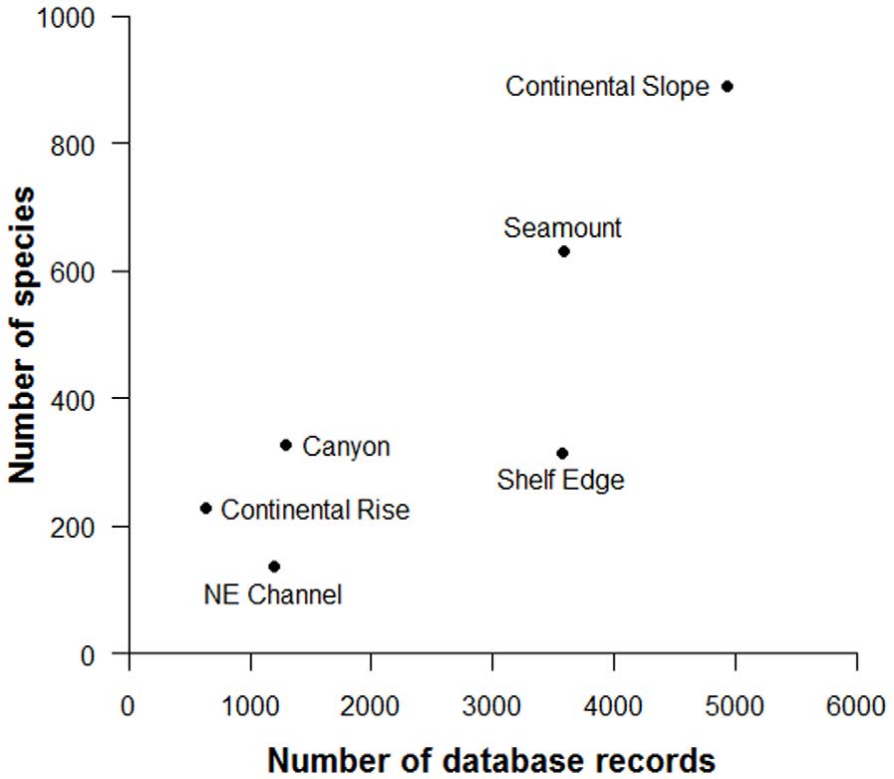
The number of database records in relation to known species. Positive correlation (Pearson's *r* = 0.86, p<0.025) between the number of records compiled within the deep-sea database (representing the number of times a sub-region has been sampled, used as a proxy for sampling effort) and the number of known species within each sub-region.

**Figure 3:**
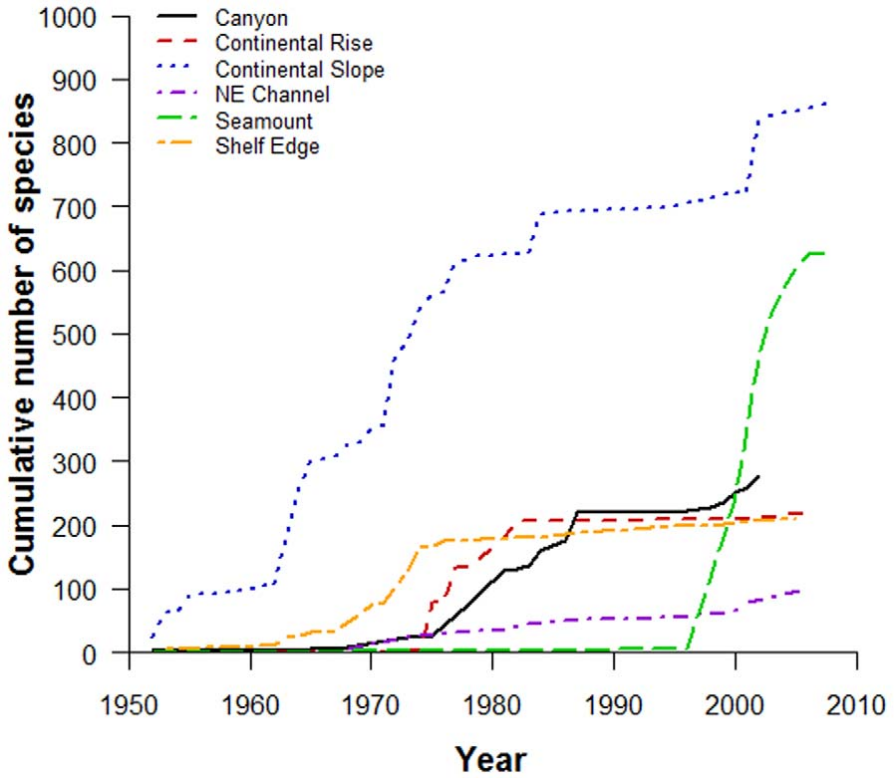
Cumulative number of known species in the deep-sea database within six sub-regions since 1950.

**Figure 4:**
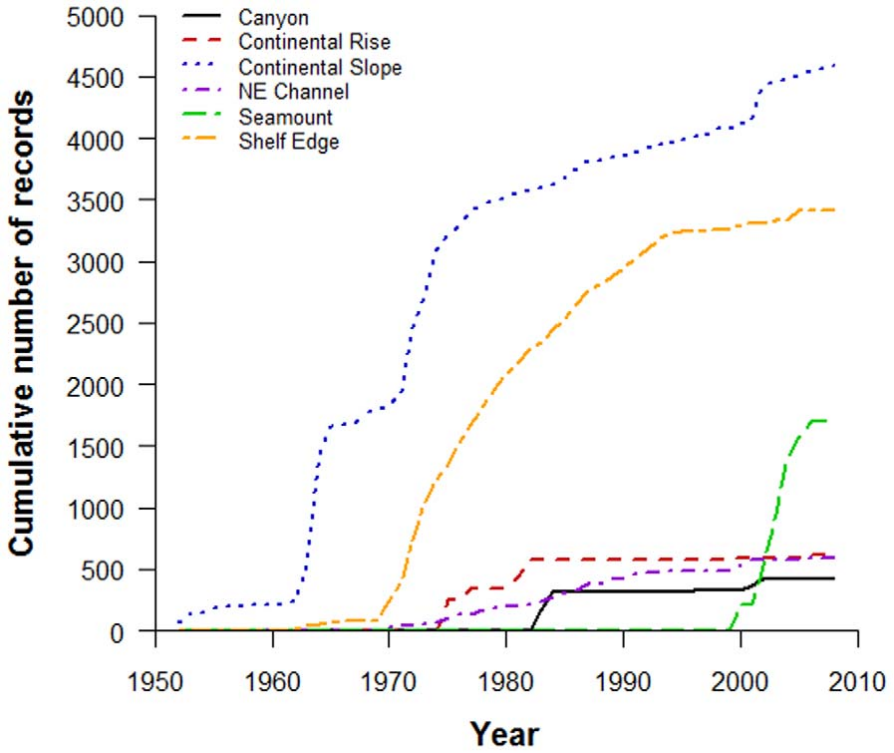
Cumulative number of species records in the deep-sea database within six sub-regions since 1950.

The number of known species in each sub-region is directly related to sampling effort. There was a strong positive correlation between the number of database records (i.e. representing the number of times a sub-region has been sampled) and the number of known species within a sub-region, such that the greater number of database records resulted in a higher number of known species (Pearson's *r* = 0.858, *t* = 3.343, *df* = 4, p = 0.014) (Table 3; Fig. 2). The rate at which species were reported (e.g. ‘discovered’) within a sub-region over time also reflects the amount of attention each sub-region has received over this same period (Fig. 3, 4). For example, the Continental Slope, NE Channel, and Shelf Edge sub-regions demonstrated a relatively monotonic increase (albeit of different magnitudes) in the number of species added to their inventories since ∼1970, related to the increasing amount of sampling occurring within these sub-regions over the same period. For the Canyon and Continental Rise sub-regions, the period 1970–1990 showed small and sporadic increases in sampling and in the number of reported species, but discovery and sampling have remained stagnant since. In contrast, the pattern of sampling and species discovery over time differs quite markedly for the Seamount sub-region; increased scientific interest since the late 1990s fostered a large and rapid increase in the number of known species within this sub-region.

Species within the database represent two Kingdoms (Animalia, Protoctista) and 18 Phyla (Table 5). Species within the Phyla Arthropoda, Chordata, Cnidaria, Echinodermata, and Mollusca occurred across all sub-regions, while the Phyla Brachiopoda, Cephalorhyncha, Echiura, and Platyhelminthes were represented by only one species occurring within only one sub-region. Other rare taxa in the deep-sea database include the Phyla Bryozoa, Chaetognatha, Ctenophora, Nematoda, Nemertina, Porifera, and Sipuncula, all with fewer than 20 species reported in any one sub-region (Table 5).

**Table 5:** The number of known species by Kingdoms and Phyla within each of 6 sub-regions.

	Sub-region					
Taxonomy	Canyon	Continental Slope	NE Channel	Continental Rise	Seamount	Shelf Edge
**Animalia**						
Annelida	58	64				12
Arthropoda	43	110	22	10	51	77
Brachiopoda		1				
Bryozoa		2				3
Cephalorhyncha		1				
Chaetognatha	2	1				
Chordata	133	357	64	154	444	100
Cnidaria	54	53	32	18	29	42
Ctenophora	3	1				
Echinodermata	21	83	9	41	38	25
Echiura		1				
Mollusca	7	152	5	4	66	32
Nematoda			2			
Nemertina					2	
Platyhelminthes					1	
Porifera	3	1	1		1	2
Sipuncula	2	3				
**Protoctista**						
Granuloreticulosa		61	1			21

A wide diversity of fishes (Phylum Chordata, Class Actinopterygii) inhabits the study region (Table 6), for a total of 647 species in 24 Orders. Species representing 9 Orders occurred in all sub-regions, while 1 Order had a single species represented in only one sub-region (Table 6). Fifteen of the 25 Orders occurred in both the Seamount and Continental Rise sub-regions, representing both mesopelagic and bathypelagic species, whereas only 11 Orders were recorded in the Northeast Channel (Table 6).

**Table 6:** The number of known species by Order within the Phylum Chordata, Class Actinopterygii, within each of 6 sub-regions.

	Sub-region					
Order	Canyon	Continental Slope	NE Channel	Continental Rise	Seamount	Shelf Edge
Anguilliformes	10	20	1	18	22	3
Aulopiformes	5	26	1	7	38	3
Beloniformes		1			1	1
Beryciformes	1	7			3	
Cetomimiformes		2			9	
Clupeiformes	2	1	3			4
Gadiformes	17	31	14	9	26	16
Lampriformes		4		1	6	
Lophiiformes	3	23	2	6	39	6
Myctophiformes	24	33		39	46	1
Notacanthiformes	1	6		3	10	1
Ophidiiformes	2	9	1	4	8	1
Osmeriformes	5	11	1	9	32	2
Perciformes	11	58	9	7	62	15
Pleuronectiformes	9	13	7	4	5	11
Polymixiiformes		1			1	1
Saccopharyngiformes		2		1	5	
Scorpaeniformes	6	18	10		10	13
Stephanoberyciformes	2	10		7	13	
Stomiiformes	18	45	7	35	81	8
Synbranchiformes					1	
Syngnathiformes		3		1	4	1
Tetraodontiformes	3	7			8	2
Zeiformes		2			2	1

The diversity of sharks and rays in the study area (Phylum Chordata, Class Elasmobranchii) was represented by 28 species in 4 Orders (Table 7). Only species in the Order Rajiformes occurred across all sub-regions. All 4 Orders occurred in the Continental Slope sub-region, while only 1 Order represented by three species occurred in the Continental Rise sub-region (Table 7).

**Table 7:** The number of known species by Order within the Phylum Chordata, Class Elasmobranchii, within each of 6 sub-regions.

	Sub-region					
Order	Canyon	Continental Slope	NE Channel	Continental Rise	Seamount	Shelf Edge
Carcharhiniformes	1	3			4	
Rajiformes	4	9	6	3	2	7
Squaliformes	2	5	1		2	2
Torpediniformes		1				

For molluscs (Phylum Mollusca), 236 species are currently reported within the deep-sea census area. Cephalopods were found in all sub-regions, while gastropods and bivalves were distributed across four sub-regions. In contrast, the Classes Caudofoveata, Polyplacophora, and Scaphopoda had <5 species occurring in only two sub-regions (Table 8). All Classes occurred within the Continental Slope, while 5 of the 6 Classes occurred within the Shelf Edge. The Seamount sub-region had particularly high species richness within the Gastropoda and Cephalopoda, but most Classes were poorly represented across the other sub-regions (Table 8).

**Table 8:** The number of known species by Class for Phylum Mollusca within each of 6 sub-regions.

	Sub-region					
Class	Canyon	Continental Slope	NE Channel	Continental Rise	Seamount	Shelf Edge
Bivalvia	4	68	1			9
Caudofoveata	1	2				
Cephalopoda	2	14	4	1	49	9
Gastropoda		62		3	17	11
Polyplacophora		1				2
Scaphopoda		5				1

We recorded 206 species of crustaceans in the Class Malacostraca. Decapod crustaceans occurred in all sub-regions, with the highest number reported on the Continental Slope and amphipod species occurred in 5 of the 6 sub-regions (Table 9). Most other crustacean Orders were only represented by a few species and were sparsely distributed across sub-regions.

**Table 9:** The number of known species by Order for Phylum Arthropoda, Class Malacostraca, within each of 6 sub-regions.

	Sub-region					
Order	Canyon	Continental Slope	NE Channel	Continental Rise	Seamount	Shelf Edge
Amphipoda	6	16	9		7	20
Cumacea	2	6				1
Decapoda	27	66	11	9	36	45
Euphausiacea	3	6			1	
Isopoda	3	7			1	2
Lophogastrida					2	
Mysida		2				
Tanaidacea	1					

For echinoderms (Phylum Echinodermata), 134 species are currently reported for the deep-sea area. Species within the Classes Asteroidea and Ophiuroidea occurred across all sub-regions, while only one species within the Crinoidea was reported to occur in the NE Channel and the Shelf Edge (Table 10).

**Table 10:** The number of known species by Class for Phylum Echinodermata within each of 6 sub-regions.

	Sub-region					
Class	Canyon	Continental Slope	NE Channel	Continental Rise	Seamount	Shelf Edge
Asteroidea	11	34	6	15	21	13
Crinoidea			1			1
Echinoidea	4	12		9	5	2
Holothuroidea	2	10		6	3	1
Ophiuroidea	4	27	2	11	9	8

A total of 104 species of anemones and corals (Phylum Cnidaria, Class Anthozoa) were present, with 2 Orders being prevalent across all sub-regions (Table 11). Two of the 8 Orders had only one species represented in one sub-region. Generally, most Orders within this Class appear to be widespread, with more than half occurring in all sub-regions (Table 11).

**Table 11:** The number of known species by Order for Phylum Cnidaria, Class Anthozoa, within each of 6 sub-regions.

	Sub-region					
Order	Canyon	Continental Slope	NE Channel	Continental Rise	Seamount	Shelf Edge
Actiniaria	9	17	11		3	10
Alcyonacea	20	15	15	10	9	12
Antipatharia				1		
Ceriantharia	2	1	1			1
Corallimorpharia		1				
Pennatulacea	5	3			3	2
Scleractinia	6	11	3	6	7	3
Zoanthidea		2	2		1	3

**Figure 5:**
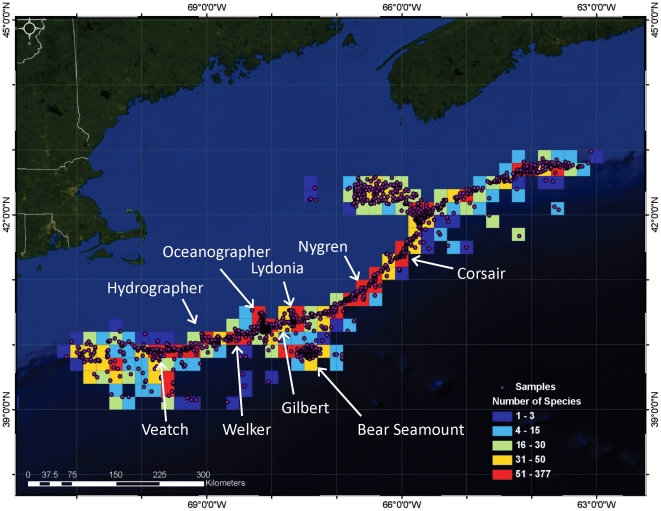
Spatial distribution of the number of known species across the deep-sea Gulf of Maine region. Grid squares are 0.2 degrees and include species records throughout the water column (from 150 m to the seafloor). Species counts are not corrected for effort or sampling method. Dots represent a species record. The names and locations of the major canyons and Bear Seamount are identified by arrows.

The spatial distribution of known species, as well as the number of records, varies across the deep-sea census area. Bear Seamount, as well as the submarine canyons (Veatch, Hydrographer, Welker, Gilbert, Lydonia, Oceanographer, Nygren, and Corsair) and surrounding continental slope along Georges Bank, and the NE Channel, appear to be zones of enhanced diversity: areas containing a high number of species (i.e. 51–377) (Fig. 5). In contrast, sections of our census area throughout the deeper Continental Slope and Rise, the western edge of the Continental Slope, and areas adjacent to Bear Seamount, appear to contain fewer reported species. The distribution of known species across the census region also appears to be related to the distribution of sampling effort, as grid squares with a low frequency of records generally have low numbers of known species. However, one exception is in the western Continental Rise sub-region, where grid squares contain few records, but >50 known species (Fig. 5).

**Figure 6:**
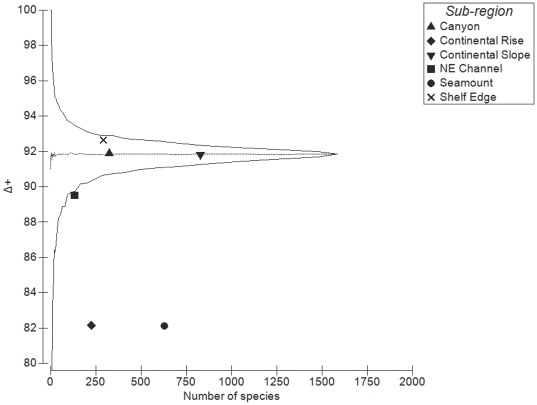
Average taxonomic distinctness (Δ^+^) by number of species for each sub-region. Central line is average taxonomic distinctness for the total list. Funnel lines are confidence limits within which 95% of simulated values lie.

**Figure 7:**
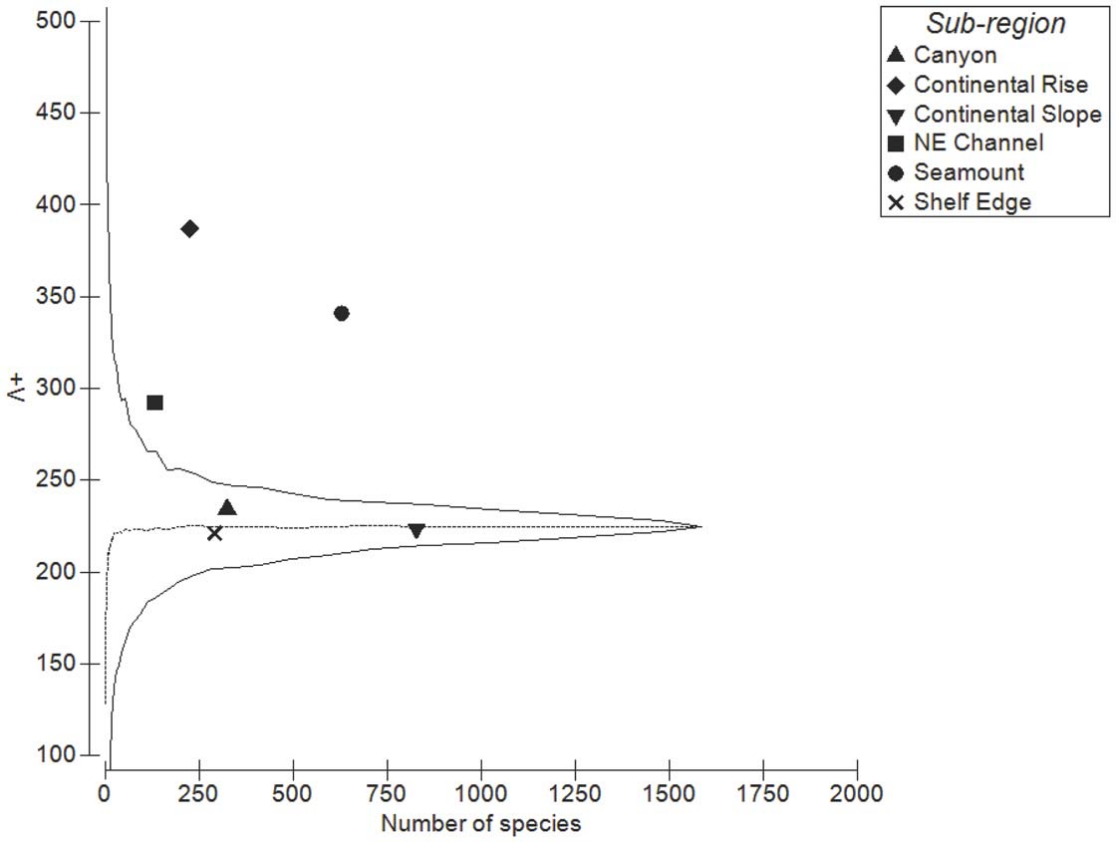
Variation in taxonomic distinctness (Λ^+^) by the number of species for each sub-region. Central line is variation in taxonomic distinctness for the total list. Funnel lines are confidence limits within which 95% of simulated values lie.

**Figure 8:**
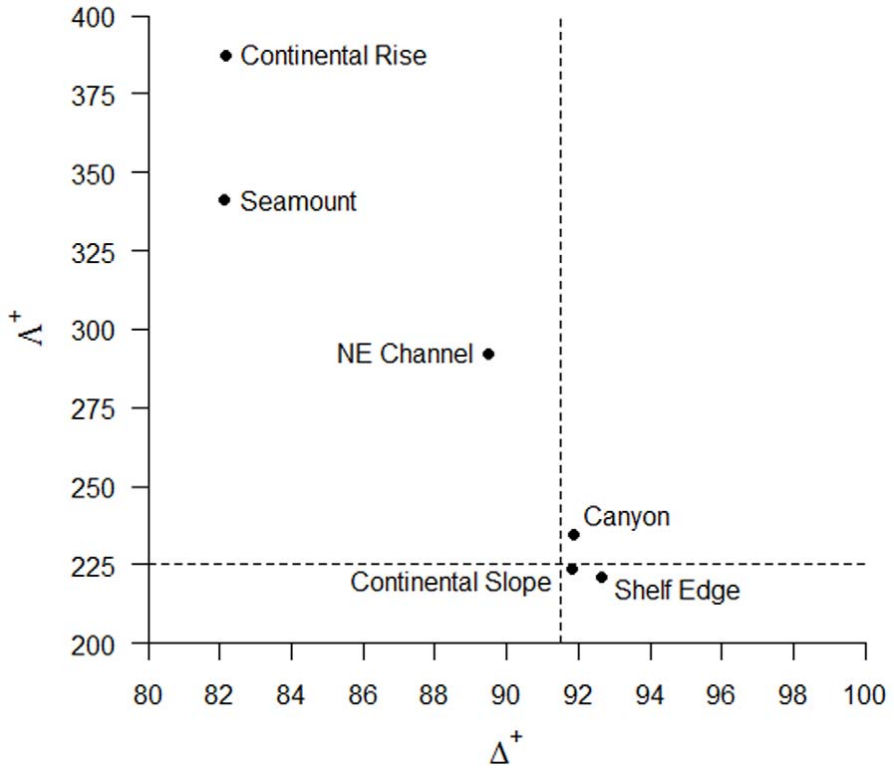
Taxonomic distinctness values by sub-region. Scatter plot of variation in taxonomic distinctness (Λ^+^) against average taxonomic distinctness (Δ^+^) values for species lists from 6 sub-regions, showing a strong negative correlation (Pearson's *r* = −0.87, p<0.05). Dotted lines denote Δ^+^ and Λ^+^ values of the master species list from Figs 6 & 7.

Values of average taxonomic distinctness (Δ^+^) deviated from expectation for three of the six sub-regions, with the Continental Rise (p<0.01), Seamount (p<0.01), and NE Channel (p<0.05) sub-regions falling significantly below expectation, while there was no significant difference in Δ^+^ between the master list and the Canyon, Shelf Edge and Continental Slope sub-regions (Fig. 6). Similarly, the Continental Rise, Seamount, and NE Channel sub-regions showed higher than expected variation in taxonomic distinctness (Λ^+^) (p<0.01), while there was no significant difference in Λ^+^ compared to the master list for the other three sub-regions (Fig. 7). Plotting the pairs of Δ^+^ and Λ^+^ values for each sub-region yielded a significant negative correlation (Pearson's product-moment correlation: *r* = −0.959, *t* = −6.823, *df* = 4, p = 0.001) (Fig. 8), demonstrating the change in both taxonomic metrics across sub-regions. Together, these results suggest that the taxonomic structure of species lists from the 6 sub-regions fall into two distinct groupings: (1) Canyon, Continental Slope and Shelf Edge have the greatest taxonomic breadth (e.g. diversity), with a wide spread of higher taxa (i.e. orders, classes) and species evenly distributed across them; and (2) Continental Rise and Seamount, and to a lesser extent the NE Channel, have lower taxonomic breadth, with many species more closely related to each other, but also some higher-level taxa with few branches (and thus family, genus, or species poor), yielding very uneven taxonomic trees.

Overall, most sub-regions were dissimilar to each other, having ≤38% similarity in species composition (Table 12). The NE Channel and Shelf Edge sub-regions had the most similar species lists, while the NE Channel and Continental Rise sub-regions had the least similar species lists. Generally, sub-regions that are geographically separated have species lists that were highly dissimilar (Seamount–Shelf Edge, NE Channel–Seamount, Shelf Edge–Continental Rise and NE Channel– Continental Rise were <10% similar), whereas sub-regions that were close geographically or physically connected shared a greater similarity among their species lists (Canyons – Continental Slope, NE Channel – Shelf Edge, and Seamount – Continental Rise had similarities ranging between 25–38%) (Table 12).

**Table 12:** Bray-Curtis similarity values (%) (based on presence/absence data) for the species lists among 6 different sub-regions.

Sub-region	Canyon	Continental Rise	Continental Slope	NE Channel	Seamount
**Continental Rise**	20.3				
**Continental Slope**	26.0	23.1			
**NE Channel**	25.1	5.5	17.2		
**Seamount**	17.8	28.0	31.4	6.5	
**Shelf Edge**	26.3	6.7	29.6	37.8	8.5

**Figure 9:**
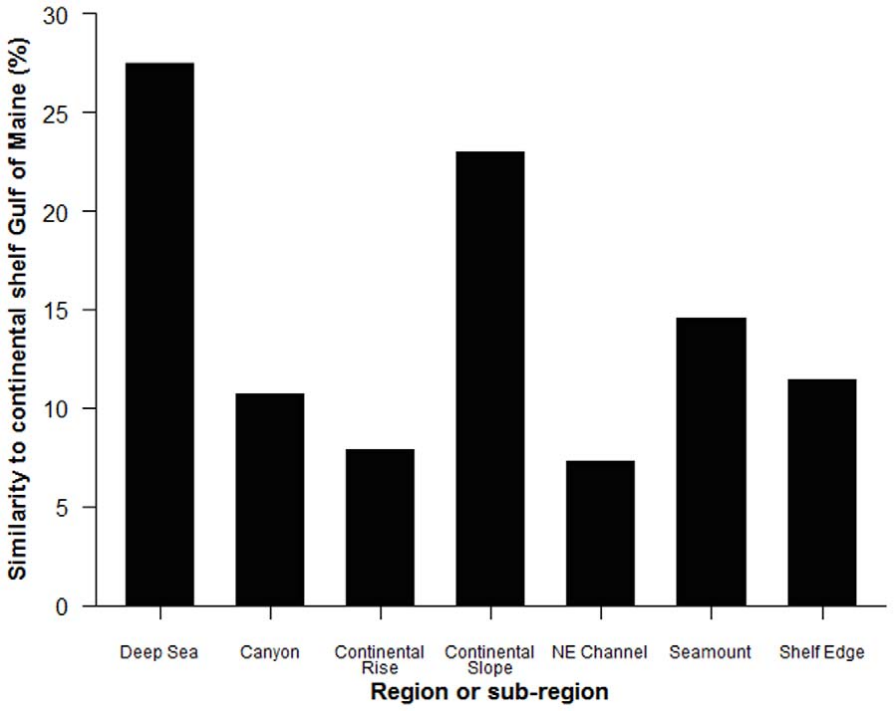
Similarity of species lists. Bray-Curtis similarity (%) of the total deep-sea region species list and the species lists for 6 different sub-regions to the continental shelf Gulf of Maine species list.

Overall, the deep-sea region and the continental shelf of the Gulf of Maine have 538 species in common with a similarity of 27.5%, while a large proportion of species (1133 species) present in the deep-sea region were not found in the continental shelf waters of the Gulf of Maine. A comparison of the similarity values of the deep-sea species lists by sub-region to the continental shelf species list demonstrates that the Continental Slope sub-region was most similar to the continental shelf, although by only 23% (Fig. 9). The species lists for the other sub-regions were less than 15% similar to the continental shelf Gulf of Maine (Fig. 9).

### Patterns of unknown biodiversity

**Figure 10:**
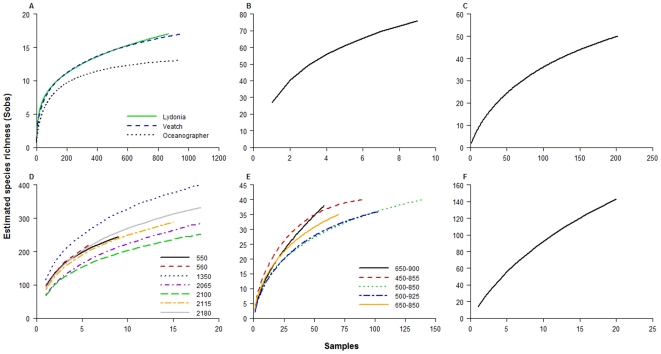
Estimated species richness (Sobs) curves by number of samples. (A) Canyon sub-region for epibenthic macro- and megafauna, as imaged in submersible photographs in Lydonia, Oceanographer and Veatch canyons in 1984 (data from [29]); Continental Rise sub-region for (B) demersal megafauna, collected from trawls using a 41′ Gulf of Mexico net between 2150–2650 m (data from [43], [44]), and (C) epibenthic and demersal macro- and megafauna, as imaged in submersible photographs between 2500–2600 m (data from Metaxas, unpublished); (D) Continental Slope sub-region for infaunal macrofauna sampled with box cores in 1983–84 at different depth stations (data from [26]); (E) NE Channel sub-region for epibenthic and demersal macro- and megafauna, as imaged in submersible photographs across different depths (data from Metaxas, unpublished); and (F) Seamount sub-region (Bear Seamount) for meso- and bathypelagic megafauna sampled using IGYPT nets, collected July 2002 (data from [30]).

Species accumulation curves for different faunal groups and sub-regions demonstrated that there remains a large portion of the deep-sea biodiversity to be discovered, although some areas appear to have been relatively well studied (Fig. 10). Most predicted species richness curves did not reach an asymptote for most faunal groups and sub-regions, particularly for mesopelagic and bathypelagic megafauna collected on Bear Seamount (Fig. 10F), for infaunal macrofauna collected at depths 550–2180 m on the Continental Slope (Fig. 10D), and demersal megafauna (Fig. 10B) and epibenthic macro- and megafauna (Fig. 10C) collected in the Continental Rise sub-region. In contrast, expected species richness curves reached or nearly reached an asymptote for benthic and demersal megafauna in the heads of three different canyons (Fig. 10A) and across several depth ranges in the NE Channel (Fig. 10E).

**Figure 11:**
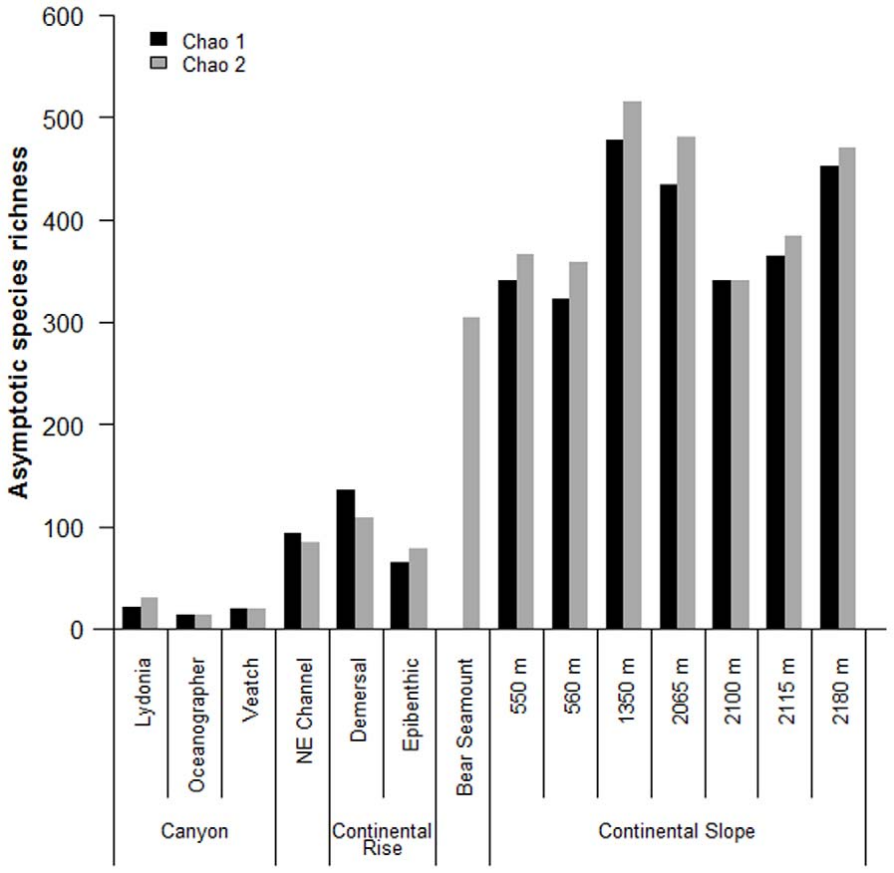
Asymptotic species richness estimates. Comparison of the asymptotic species richness estimates (Chao 1 and Chao 2) for 5 different sub-regions in the deep-sea region of the Gulf of Maine. The Chao 1 estimate for Bear Seamount could not be calculated due to lack of singletons within the data.

The completeness of our taxonomic inventories ranged from ∼55–100%, and varied by sub-region and faunal type (Table 13). Based on both Chao 1 and Chao 2 estimators, taxonomic inventories of benthic and demersal megafauna within the Canyon sub-region were the most complete (58–100%), while the Seamount mesopelagic and bathypelagic megafauna was only 47% complete based on the Chao 2 estimator (Table 13). For most sub-regions, both Chao 1 and 2 yielded similar estimates for true species richness (Fig. 11). Based on these estimators, the number of remaining species to be discovered within the deep sea region of the Gulf of Maine ranges from 0–13 for megafauna in Canyons and 76–197 for macro-infauna on the Continental Slope (Table 13).

**Table 13:** Estimates of the unknown biodiversity in the deep-sea system of the Gulf of Maine.

Sub-region	Subset	Observed species	Chao 1	Percent complete	Species remaining	Chao 2	Percent complete	Species remaining
Canyon	Lydonia	17	21	81.0	4	30	57.6	13
	Oceanographer	13	13	100	0	13	100	0
	Veatch	17	20	85.0	3	20	85.0	3
NE Channel		69	94	73.8	25	84	82.2	15
Abyssal	Demersal	76	136	55.8	60	109	69.9	33
	Epibenthic	50	64	73.8	25	78	82.2	15
Bear Seamount*		143	–	–	–	304	47.1	161
Continental Slope	550 m	245	340	72.1	95	366	66.9	121
	560 m	225	323	69.7	98	359	62.7	134
	1350 m	401	477	84.1	76	515	77.9	114
	2065 m	283	434	65.2	151	480	59.0	197
	2100 m	253	340	74.4	87	340	74.4	87
	2115 m	287	365	78.6	78	385	74.5	98
	2180 m	332	452	73.5	120	470	70.6	138

Percent completion of species lists and the number of species remaining to be ‘discovered’ for subsets of sites, depths, or faunal types for five different sub-regions. See Table 2 for a summary of the data sources used in this analysis.

*No Chao 1 estimate exists for Bear Seamount due to a lack of singletons within the data.

## Discussion

### Known and unknown biodiversity

Through an extensive survey of existing studies, databases, museum records, and unpublished information (where possible), we present the first estimate of diversity for the deep-sea Gulf of Maine region: 1671 species, distributed across 2 Kingdoms and 18 Phyla. Unsurprisingly, the most frequently reported species within the region were megafaunal species of corals (Cnidaria/Anthozoa), fishes (Chordata/Actinopterygii) decapod crustaceans (Arthropoda/Decapoda), and echinoderms (Echinodermata), likely because of their large body size, high commercial or scientific interest, relatively high frequency of directed sampling, and/or frequent presence as by-catch. In contrast, taxa with smaller body forms, typically comprising the infaunal and/or meiofaunal groups, and/or with little to no commercial or economic interest, and that are relatively difficult to sample and/or identify, were infrequently reported and remain poorly known: Brachiopoda, Cephalorhyncha, Echiura, Platyhelminthes, Bryozoa, Chaetognatha, Ctenophora, Nematoda, Nemertina, Porifera, and Sipuncula. Lack of knowledge for these taxa is not exclusive to the deep sea, and is a pattern often observed in many marine areas, likely due to the small size of the taxonomic community available to study them [50], [51]. In addition, there is a paucity of information on the life-history characteristics, dispersal patterns, modes of reproduction, and recruitment patterns for almost all species. The difficulty of quantifying diversity patterns for these poorly-studied taxa is further exacerbated by the lack of widespread general sampling in the deep-sea region.

The number of known species within each sub-region is related to the frequency with which it has been sampled. Our relatively greater knowledge of diversity along the Continental Slope sub-region likely reflects sampling effort, as it is a large area that has been frequently targeted by commercial and scientific trawling. While true species richness will differ among sub-regions due to differences in hydrodynamics, habitat characteristics and spatial extent, and variation in the strength of biological interactions (e.g. differences in predation, competition, facilitation, etc.), several sub-regions do appear to be under-sampled compared to the others (particularly the NE Channel and Continental Rise), potentially leading to an underestimate of the number of known species in these areas. For example, 44% of the known species in the NE Channel have been recorded since 2000 (see section ‘Spatial distribution and drivers of biodiversity: The NE Channel’ below). Continued scientific interest in sampling the benthic macro- and megafauna (e.g. corals and associated organisms), as part of the Canadian Discovery Corridor project (www.marinebiodiversity.ca/cmb/research/discovery-corridor) and the Canadian Healthy Oceans Network (www.marinebiodiversity.ca/CHONe), will shed more light on deep-sea macrofaunal community structure and likely lead to many new discoveries in this sub-region in the next decade.

Chao metrics tend to underestimate true species richness and can be considered a minimum bound on our species richness estimates [52], indicating that the deep-sea census region could have up to 50% of its species inventory remaining to be discovered. Estimates of species richness calculated in this study were for macrofaunal and megafaunal species, and virtually no data were available for meiofaunal organisms, precluding the estimation of species richness, and thus the number of species remaining to be discovered, for this faunal type. Giere [51] states that the diversity of deep-sea meiobenthos is unexpectedly high; sampling of the deep-sea benthos has yielded 40 species of foraminiferans per cm^2^, and an average of 25–50 distinct species of nematodes or harpacticoids per 100 individuals of meiofauna. Given the large unknowns surrounding estimates of meiofaunal diversity in the system, as well as for mega- and macrofaunal species, it is clear that the total amount of biodiversity in the deep-sea system of the Gulf of Maine remaining to be discovered is much higher than 50%.

The lack of taxonomic expertise for species-level identifications remains a major limitation to advancing our knowledge of biodiversity in the deep-sea Gulf of Maine region. Many of the studies (both published and unpublished) from which we drew data lacked a detailed taxonomic identification for specific groups, precluding the use of such information in most of our analyses. For example, non-decapod crustacean groups, such as tanaids, isopods, amphipods, cumaceans, and harpacticoids, are frequently only reported to Class or Order in many studies (e.g. [53]). Within the database, of the number of records of individuals in the phyla Annelida, Nematoda, and Porifera, 30%, 51%, and 95% of individuals, respectively, were not identified past the family level. The number of Annelids (as well as macrofauna from the continental slope in general) reported herein for the deep-sea region should be much higher; in a quantitative study of soft sediment habitats on the continental slope of Georges Bank, Maciolek et al. [26] found that out of a total of 1019 species in 191 box core samples, annelids accounted for 45% of all species within the infaunal community. Formal species names for deep-sea nematodes are often unavailable, and researchers are often restricted to classifying individuals based on functional forms or morphology (e.g. [54]), while studies focussing on sponges often rely on colour and/or form as an index (e.g. [55]), but the extent to which these descriptors relate to species richness is currently unknown. Advances in molecular techniques may provide a way forward to quantifying some of this unknown biodiversity, particularly for small-bodied species (e.g. <1 mm), as well as prove invaluable for identifying cryptic species and early life-history stages (e.g. larvae).

### Biodiversity structure

Compared to the remainder, the Continental Rise and Seamount sub-regions displayed distinct patterns of biodiversity structure, as they both demonstrated lower taxonomic breadth (i.e. diversity) (Δ^+^) and greater than expected variation in taxonomic distinctness (Λ^+^) in their species lists. These results suggest that these two sub-regions harbour distinct faunal assemblages that are more closely related taxonomically within them than to any other sub-region. However, the Continental Rise and Seamount sub-regions are also dominated by only 5–6 phyla, resulting in the lower than expected Δ^+^ values. The negative correlation between Δ^+^ and Λ^+^ implies that changes in benthic topography (smooth and flat to rugged and variable; from the Continental Slope to Canyons and NE Channel) and increasing geographic distances from the continental shelf of the Gulf of Maine (from the Shelf Edge to the Continental Rise and Seamount) are associated with the loss of higher taxa (reduced Δ^+^). Additionally, the higher taxa that are present are represented by only a few species, genera or families, creating an unbalanced tree (increased Λ^+^). Decreasing diversity with increasing distance from continental shelf waters may be related to changes in food supply (e.g. [56]). However, the presence of cryptic species within these sub-regions, as well as inconsistencies in the definition of taxonomic units across multiple phyla, may also affect the estimates of Δ^+^ and Λ^+^ (see [46] for further discussion on limitations of taxonomic distinctness analyses). This changing pattern of biodiversity structure may also reflect the degree to which these sub-regions have been sampled, as the Continental Slope and Shelf Edge have had a longer history of sampling and species discovery than the Continental Rise and Seamount sub-regions. Future sampling within the region and across multiple habitats will aid in validating or rejecting these hypotheses.

Similarity in species lists of different sub-regions decreased with increasing geographic separation (e.g. NE Channel and Seamount). The similarity between the continental slope and seamount fauna is likely driven by the presence of mobile species (e.g. fish, cephalopods) found in both regions. A lack of similarity suggests little overlap in species distributions and weak long-distance (>100–1000 km) dispersal of propagules among sub-regions in the deep-sea region of the Gulf of Maine. For benthic fauna, the weak currents typical of continental slope areas [57] may prevent the long distance dispersal of larvae, reducing diversity among geographically separated areas within a sub-region (e.g. Continental Slope, Canyons) or among sub-regions. For example, species inhabiting canyon environments may easily disperse with the strong currents moving from the canyon head to mouth and vice versa [58], but dispersing in an across-slope direction into an adjacent canyon may be unlikely. In contrast, the periodic intrusions of Gulf Stream warm-core rings at mid-slope depths (600–1000 m) may increase dispersal distances of fauna, if propagules are available at the right time (i.e. a dispersal lottery). However, this similarity pattern may also reflect the specific habitat requirements of the abundant or dominant species within the region. For example, corals and other suspension feeders require hard substrates for attachment and strong currents for the delivery of food particles, and thus dominate in canyons [27], Bear Seamount [59], and the NE Channel [18], where both habitat characteristics are typical of the benthos.

Based on the low similarities in faunal composition among sub-regions, the deep-sea census region appears to be mostly distinct from the continental shelf of the Gulf of Maine. While some overlap in diversity exists, this overlap does not generally extend beyond the Continental Slope sub-region. For example, the Shelf Edge, Canyons, and NE Channel, all had species lists which were <12% similar to the continental shelf. Given the circulation patterns in the Gulf of Maine, these sub-regions, particularly the NE Channel, are the main corridors that link the deep-sea and shelf Gulf of Maine regions, and thus it is reasonable to hypothesize that similarities among their fauna would be high. Thus, their distinct faunal communities may be indicative of a boundary that may occur at some depth along the Continental Slope-Shelf Edge-NE Channel continuum, separating the deeper habitats from the shallower areas. The presence of a permanent thermocline at 200–600 m depths may act as a physiological boundary to dispersing or mobile species or may limit the transport of propagules. In addition, the variations in local topography at the shelf-slope break, such as the transition from soft to hard substrates, may restrict species of the continental shelf of the Gulf of Maine from extending their range into the deeper areas of the continental margin. However, the lack of a higher degree of overlap between the shallow and deep regions of the Gulf of Maine may also be due to under sampling of the deep-sea region rather than a true difference in taxonomic composition between areas. For example, a recent study by Thoma et al. [60] demonstrated genetic connectivity within coral taxa distributed between western New England seamounts, submarine canyons and a deep Gulf of Maine basin.

### Spatial distribution and drivers of biodiversity

‘Hotspots’ of biodiversity are defined as ‘centres of endemism’ or regions housing high concentrations of endemic species [61]. While we have not found any evidence of endemics within any of the sub-regions in the deep-sea census area, there does appear to be some degree of separation among faunal assemblages by sub-region. Despite these limitations, the spatial distribution of diversity across the study region suggests that several areas, particularly Bear Seamount, the NE Channel, and several Submarine Canyons (e.g. Hydrographer, Oceanographer, Lydonia, and Veatch) and surrounding Continental Slope, are zones of enhanced diversity. These areas appear to harbour the greatest numbers of species of corals, anemones, fish, echinoderms, crustaceans, and cephalopods across the region. The following sections summarize what is known about these specific sub-regions and highlight the biotic and/or abiotic factors driving biodiversity within them.

#### Bear Seamount

Bear Seamount (39°55′N, 67°30′W) is the oldest and western-most seamount in the New England Seamount Chain (NES), and is the only seamount included in the deep-sea Gulf of Maine census area. Bear Seamount formed about 100 million years ago by the Great Meteor/New England hot spot [62]. It rises from the continental slope at depths of 2000–3000 m to a generally flat summit at 1100 m depth, and is influenced by three currents: the Gulf Stream and associated eddies, the Deep Western Boundary Current and the Antarctic Bottom Water at the base [19], [24]. Bear Seamount is within the United States' Exclusive Economic Zone (EEZ) and several potentially exploitable fish, cephalopods and crustaceans have been collected there [19]. Only one known exploratory commercial fishing cruise has occurred at Bear [19].

To address the lack of biotic sampling at Bear Seamount, the National Oceanic and Atmospheric Administration (NOAA) National Systematics Laboratory and the R/V DELAWARE II began a program of exploratory trawling to document the meso- and bathypelagic fauna associated with the seamount [19], [38], [63], [64]. Additionally, in 2003–2005, NOAA's Oceans Exploration program funded several expeditions to Bear (and other northwest Atlantic seamounts) for studies of deep-water coral communities and associated fauna. As a consequence of these expeditions, Bear Seamount is now comparatively well-sampled and characterized from a biological and physical perspective, although species accumulation curves suggest more taxa may still be discovered. Coral assemblages appear to be stratified by depth [65], but local endemics have not been found [60]. Several fish species found at Bear are rare in the northwest Atlantic, and some are only known from the eastern Atlantic [19], [64], [66]. These species may represent a natural enlargement of the range (“natural invaders”; [30]) or they may be relict populations that use the seamounts as a refuge [67]. New species continue to be described (e.g. [63], [68], [69]) and commensal relationships discovered [65], [70], [71].

Our preliminary results across multiple phyla show that Bear Seamount has a high diversity of corals, anemones, fish, echinoderms, crustaceans, and cephalopods compared to other areas within the deep-sea Gulf of Maine region. The low Δ+ suggests taxa are closely related to each other (i.e., there is a short average taxonomic path length between any two taxa). The very high Λ+ suggests the hierarchical organization of taxa found is extremely uneven, with multiple species within some genera mixed with monotypic families. These two metrics are consistent with the sampling programs described where the datasets for some phyla are more accessible than others.

#### Continental Slope

Patterns of mega- and macrofaunal abundance and diversity on the continental slope have been documented to vary with depth [15], [26], [72]–[74]. Hecker [15] identified four megafaunal zones on the continental slope, with the boundaries being marked by abrupt shifts in faunal density. Highest densities of megafauna were found in the upper (200–500 m) and lower slope (>1600 m) while lower density zones occurred on the upper-middle slope (500–1200 m) and the transition zone (1200–1600 m) [15]. For macrofauna, Maciolek et al. [26] found that upper slope stations (255 m and 550 m) were dominated by filter feeders, while mid-slope (1220–1350 m) and deep slope stations (2100 m) were dominated by deposit feeders. While macrobenthic density and biomass appear to decrease with increasing depth [26], [72], diversity appears to be maximal at mid-slope depths (1220–1350 m), as compared to shallower (255–550 m) and deeper (2100 m) depths [26], [74].

The pattern of species diversity and distribution with depth on the continental slope adjacent to the Gulf of Maine is controlled by the effects of local topography on currents and accompanying effects on sediment grain size and food availability. Below ∼300 m, the continental slope is predominantly comprised of fine silt and clay-sized particles [13], which are vulnerable to resuspension and removal. Higher current intensities and more frequent resuspension events occur on the upper and lower slope than on the middle slope [75], thus the finest-textured sediments are found on middle slope (∼1250 m) while coarser sediments are found on the upper (250 and 550 m) and lower (2100 m) slope [26]. The rarity of strong near-bottom flows in the middle slope, resulting from its greater steepness, enhances the accumulation of fine-grained sediment and phytodetritus, enhancing the diversity of infaunal suspension and deposit feeders [75]–[77].

Natural and anthropogenic disturbances have a negative influence on species diversity on the continental slope. In recolonization experiments off Georges Bank, Maciolek et al. [26] found that after 14 months, densities of macrofauna in experimental sediment trays remained an order of magnitude lower than in undisturbed sediments, suggesting that recovery rates of defaunated sediments to be very slow and can last on the order of years. Natural events such as benthic storms [53] and periodic currents associated with Gulf Stream warm-core rings [58] erode sediments from the seafloor, reducing the physical substrate heterogeneity provided by tubes, burrows and mounds. Increased fishing pressure in the region has increased the frequency of severe disturbances [78], [79], which disrupts the structure and diversity of benthic communities on the continental slope [80].

#### Submarine Canyons

Most information on patterns of biological diversity and structure of benthic canyon faunal communities in the region come from the best studied canyons: Lydonia, Veatch, Hydrographer, and Oceanographer [12], [16], [26]–[29], [42]. Three distinct megafaunal zones are evident within canyons (i.e. shallow, middle and deep), with the boundaries between zones occurring at ∼400 m and 1100 m [12], [27], [28]. Highest diversity is found in the middle-depth zone, while faunal densities are generally highest in the shallow zone, and decrease with depth [12], [27]. The composition of the shallow zone assemblage is typically the most variable and is dominated by taxa preferring soft substrates, while the middle depth zones are dominated primarily by sessile filter feeders and other fauna associated with hard substrates [27], [28]. Deposit feeding predominates in the deep zone. The overlap in common fauna between adjacent zones suggests species are gradually replaced along the depth gradient [28].

The high diversity and abundance of benthic faunal communities in submarine canyons is primarily linked to variations in surface geology, sedimentary features, and currents, which provide distinct habitat types that can support a wide variety of organisms [12], [28], [29], [81]. Habitats that are favourable sites for burrowing, primarily clay and siltstone outcrops, attract large numbers of epibenthic organisms and demersal fishes, and become so inundated with biologically eroded excavations of various shapes and sizes that they resemble a “Pueblo Village”, providing a three-dimensional shelter for ∼20 different species [12], [16], [29]. Areas of glacially rafted boulders also provide deep crevice habitats for burrowing megafauna [29]. Currents are higher than on the surrounding slope, rendering canyons areas of active erosion and conduits for the channelling of material from the upper shelf to the continental rise and abyss [58]. This allows the delivery of high concentration of food particles necessary to sustain the observed large populations of suspension feeders [27], zooplanktivores [82], and near-bottom scavengers [81] observed.

Species diversity and abundance are higher in canyons than at comparable depths on the slope [16], [26]–[28]. At depths between 400–1100 m, Hecker et al. [27], [28] found the slope fauna was dominated by the crab *Chaceon quinquedens* and demersal fish, while assemblages in canyons at these depths were comprised by small shrimp species, and sessile filter feeders such as corals and sponges. However, megafaunal assemblages within submarine canyons were also less cohesive than on the slope at comparable depths [28]. At 550 m depth, Maciolek et al. [26] found both macrofaunal and polychaete abundances to be higher within rather than outside Lydonia Canyon. These differences have been attributed to the addition of trophic types unique to canyon habitats, as well as differences in current regimes and disturbance rates between canyon and non-canyon habitats. The high heterogeneity of substrates within canyons, as compared to the homogeneous substrates on the slope, support high megafaunal diversity by providing attachment sites for sessile filter-feeding fauna and spatial heterogeneity of sediment types for other fauna [27]. Differences in sediment composition, which relate to differences in the strength and direction of currents, as well as disturbance by large megafaunal species present on the slope, may also be driving the differences in macrofaunal diversity and composition between canyon and non-canyon habitats in this region [26].

#### The NE Channel

NE Channel harbours the densest populations of deep-water corals in the region, most likely because of its physical characteristics. It is comprised of three steep canyons that drop into depths of 1000 m, bound by flat sandy bottoms. In terms of circulation, water flowing along the slope enters at the northeastern edge of the channel, and exits at the southwestern edge [21], [22]. The location of NE Channel at the opening of the Gulf of Maine into the NW Atlantic, in combination with its glacial origin and the steep walls of the canyons, result in local acceleration of the currents, and low and patchy sediment accumulation, with much of the substrate being pebble, cobble, boulders and rocky outcrops [17], [18]. These two habitat characteristics allow for the presence of dense populations of suspension feeders (such as deep-water corals, sponges and echinoderms) that require hard substratum for attachment and strong currents for the delivery of high concentrations of food particles.

The density of both corals and other macro- and megafauna increase with depth, presumably because of the decreasing disturbance in terms of fishing activity [18], [31], [36]. Several species of anthozoans, polychaetes, echinoderms, bryozoans, and sponges are present in higher abundance in NE Channel than in surrounding locations, both in areas with and without coral. Several fishes (particularly *Sebastes* spp.) tend to aggregate near boulders (with and without coral), presumably to reduce energy consumption by avoiding swimming and resting near the bottom where currents are slow. A few symbioses between corals and crustaceans and ophiuroids have been documented, although the degree of obligatory association remains less clear [83], [84]. Significant aggregations of suspension-feeding ophiuroids form extensive beds throughout the channel [35]. Despite this elevated faunal concentration in NE Channel, substrate does not appear to be limiting and possible competition for space appears unlikely since un-colonized boulders are common, and several species often co-occur on the same boulder [18], [31]. Consequently, a major regulatory factor of the epifaunal assemblages is most likely larval supply and recruitment. Studies are in progress that attempt to document the rate of colonization of benthic invertebrates in these rich assemblages (Metaxas, unpublished data).

### Pressing questions and research needs

The current level of knowledge of biodiversity in the deep-sea Gulf of Maine region is still rudimentary. Our ability to synthesize our understanding of this ecosystem to answer basic and applied questions is hampered by the lack of sufficient data for many taxonomic groups, which stems from three main constraints: sampling biases/issues, life-history characteristics of target species, and the lack of trained taxonomists, especially in economically unimportant groups. Sampling in this region is still mostly exploratory and usually concentrated in an area of particular interest (e.g. seamounts, NE Channel), which leads to improved knowledge of known species or habitats; however, a greater effort is needed to improve our knowledge of ecological processes driving patterns of diversity. All sampling to date has generally been descriptive; the lack of standardized time-series sampling to understand the dynamics of abundance and distribution of most groups, as well as the lack of experimental manipulations, has made comparisons of patterns of diversity among different habitats (e.g. slope, seamount, canyons, etc.) or understanding the relative roles of abiotic and biotic drivers in structuring diversity, problematic. Different sampling approaches and use of different sampling gears can provide a different view of assemblage structure and biodiversity patterns (e.g. [42]). Our knowledge of diversity is primarily restricted to adults, as current sampling efforts primarily target these stages. Data on recruitment, dispersal, and connectivity among populations within and among sub-regions for most taxonomic groups is severely lacking. Lastly, a lack of taxonomists with deep-sea expertise remains a major impediment to moving forward.

These constraints lead to many remaining and unanswered scientific questions for the deep-sea Gulf of Maine region. Quantifying patterns of variation in space and time is essential to detecting and/or predicting changes in the abundance and diversity of species, communities and ecosystems in the future. Future work should also examine the faunal composition and how species interactions structure deep-sea communities, as well as the role of physico-chemical processes in mediating diversity. In addition to these fundamental questions, there also remain many broader-scale questions involving the relationship of the deep-sea Gulf of Maine region to the surrounding ecosystems. Watling and Auster [32] documented the presence of several species of deep-water corals in both the Gulf of Maine basins and the continental slope and associated canyons, leading to questions regarding the linkages and magnitude of exchange between the deep-sea and shelf communities and whether such patterns hold for all taxa. Moore et al. [30] identified 17 different fish species at Bear Seamount and the continental slope whose next nearest known occurrences were 1000 km away, leading to questions regarding dispersal corridors along the NW Atlantic that may link the continental slope and seamount habitats with the eastern and western Atlantic.

Gaining a more complete picture of the deep-sea communities in the Gulf of Maine region at present is critical to detecting and monitoring future changes. Developing a baseline estimate of diversity is absolutely imperative, particularly in light of the future potential anthropogenic impacts in the deep-sea Gulf of Maine region: offshore waste disposal, chemical contamination, expanding oil and gas exploration, alien species, increased ship traffic, fishing to progressively deeper waters, and climate change [2], [85]. Given these expanding threats to the deep-sea Gulf of Maine region, further knowledge of diversity patterns will also aid in planning management strategies, designing MPAs, and contributing to inter-governmental approaches to ecosystem-based management. For example, a coral conservation plan was developed for the Canadian Maritimes administrative region of Department of Fisheries and Oceans (DFO) by the Eastern Scotian Shelf Integrated Management Initiative (ESSIM) with several conservation, management and research objectives [86]. The general purpose of the plan was to document existing coral conservation efforts and present a comprehensive approach, identify future research needs in coral biology and ecology, and build collaborations amongst the various stakeholders [86]. The NE Channel Coral Conservation Area (NECCCA) was established in 2002 by DFO because it contains the highest known density of intact large octocorals (*Paragorgia arborea* and *Primnoa residiformis*), and is one of three locations where coral conservation is focussed in this region (e.g. [87]). The Northeast Channel is an important fishing area targeted by otter trawls, longlines and gillnets and increased signs of fishing impact were discovered by surveys done by DFO and Dalhousie University (Halifax, NS, Canada). As a consequence a 424 km^2^ area was set aside for conservation which extends to 1200 m depth and encompasses two zones: a restricted fishing zone (90% of the area) and a limited bottom fishing area (the remaining 10%).

### Technology and future sampling

Knowledge of the diversity of deep-sea ecosystems would have been limited without recent improvements in technology. For example, human-occupied submersibles and remotely operated vehicles (ROVs) have allowed the extension of our knowledge by permitting detailed and precise sampling of the distribution of organisms across deep-sea landscapes, as well as collection of individuals for taxonomic identification. However, as biodiversity research moves into the post-CoML era, several areas must continue to be addressed and improved. We need to increase our capability for sample collection, preservation, time-series monitoring, and for experimental manipulation. All biodiversity studies will be compromised if the decline in training taxonomists persists. Reversing this trend with attention to blending taxonomic skills with technology skills should be a common underlying goal of future collaborative efforts. Identifications, photographs and videos should be properly credited with extensive metadata. Three areas in need of technological innovations and recommendations for future sampling involve improving the accuracy of species-level identifications, making such information more rapidly available, and conducting large-scale repeatable analyses of deep-sea biotic data.

Technological innovation that facilitates rapid, confident identification of species in the field is critical to improving knowledge of diversity, and could be easily implemented using five key approaches: taxonomic expertise, field guides, identifications from video, vouchering and documentation, and in-field DNA analyses. Exploratory cruises should have taxonomic specialists on board or on retainer. In the case of Bear Seamount the presence of multiple taxonomists resulted in highly reliable field identifications and expedited the finalized species lists. Creating traditional field guides to deep-sea taxa would facilitate on-board identifications. Color photographs taken at the time of capture provide essential documentation of ephemeral features, such as photophore color for cephalopods. Additionally, re-focusing on populating existing web-based specialist taxonomic sites that can be field accessed provides two benefits. First, it makes more taxonomic detail available than what is traditionally found in field guides (e.g. Tree of Life at http://tolweb.org/tree?group=Cephalopoda), and second, content from these sites is harvested and re-packaged by other websites (e.g. EOL) and disseminated to a larger, popular audience. Videotaping of transects in high resolution formats yields enormous quantities of taxonomic and distribution data, especially of benthic environments, but requires considerable post-cruise analysis. Species-level identifications are difficult due to the lack of morphological detail available from video, especially of highly mobile neritic species. For mid-sized to small sampling programs without dedicated post-cruise analysis capabilities, this remains a major stumbling block. Specimens that have been sampled for DNA analysis must be routinely photographed and deposited in a regionally or taxonomically appropriate museum. Vouchering allows verification of the present study, and cost savings for future studies that may rely on these specimens in lieu of new collecting. Personnel and materials costs for vouchering and documentation must be included in funding requests. As DNA analysis becomes more commonplace, it is becoming possible to have on-site molecular labs to assist in field identifications and preservation decisions.

To increase the availability of biodiversity information, identifications, station data and physical data recorded at time of sampling must be linked and made available to the larger scientific community as quickly as possible. Immediate, on board database recording of specimen information linked to shipboard geo-referencing information is critical. For example, the Bear Seamount cruises [19], [30] immediately input data into the NOAA's Fisheries Scientific Computing System (FSCS) and the database was quickly accessible and ready for mining by the scientific community. Database development specific to deep-sea collecting would be a useful advancement. The immediate curation of new/all specimens should be encouraged, including all geo-referencing information and an accompanying photograph where possible. Development of museum cataloguing systems that can interface with shipboard data collection would facilitate this activity. Including costs for curation and storage in the original funding requests is imperative and will facilitate rapid availability of information. In addition, large scale data depositories such as OBIS, GBIF and Seamounts Online (http://seamounts.sdsc.edu), should receive and archive data as soon as it is quality checked and the main results have been published in the peer-reviewed literature.

Large-scale repeatable analyses of deep-sea biotic data are also crucial to developing a broad understanding of deep-sea diversity patterns. Economically important species have long been collected in comprehensive, statistically significant sampling programs (see ECNASAP, NEFSC, and VIMS datasets); non-commercially important species should be dealt with similarly. Single sampling events are important for recording biodiversity, but are not sufficient to understand the processes which drive ecosystems. Replicate samples at multiple depths taken day and night at Bear Seamount [19] provide a statistically sound framework for future analyses. Funding repeat sampling events over multiple years in multiple seasons is crucial. Use of GIS for spatial analysis is common in terrestrial systems, but still growing in marine systems. Deep-sea maps and biotic and abiotic coverages should be developed for community use (e.g. benthic cover, physical characteristics, species distributions, and previous sampling activities) and software applications to handle volumetric analyses are needed.

Over the coming decades, the application of both existing and new technologies would greatly improve our knowledge of diversity in Gulf of Maine deep-water habitats. For example, having a ROV or submersible permanently dedicated to the east coast region would facilitate the rate at which we study deep-sea diversity, discover new habitats, conduct manipulative experiments, and allow for a greater frequency of *in situ* observations of critical or sensitive habitats. While deep-sea exploration is inherently expensive, we will need to balance funding opportunities to dedicate effort to the exploration of new sites with repeat visits to existing areas of interest. The implementation on the east coast of a cabled seafloor observatory, such as the NEPTUNE (North-East Pacific Time-Series Underwater Networked Experiments; www.neptune.washington.edu/ and www.neptunecanada.ca) or VENUS (Victoria Experimental Network Under the Sea; http://www.venus.uvic.ca/) observatories that use high-speed fibre optic telecommunication technology to create a permanent link to monitoring instruments deployed across a broad spectrum of undersea environments, would provide a wealth of new information and would open up new frontiers into the monitoring of various habitats across the shallow and deep-sea region of the Gulf of Maine and allow experimentation in these habitats over both short (seconds–months) and long (years–decades) timescales.

### Conclusions

Despite decades of scientific study, our knowledge of the biodiversity of the deep-sea continental margin bordering the Gulf of Maine remains limited. Our efforts to synthesize biodiversity knowledge and patterns have revealed that the vast majority of deep-sea sub-regions are under-sampled, and previous sampling efforts have been highly variable over both spatial and temporal scales. Information on the life-history characteristics, dispersal patterns, modes of reproduction and recruitment patterns of most species in this deep-sea region, particularly for non-economically important ones, is also severely lacking. Despite the lack of data, we have herein created a baseline estimate of diversity for the region, which will aid in the monitoring and detection of future changes occurring in the system in future. We have identified areas of high structural complexity and topographic relief (the NE Channel, Bear Seamount, and several submarine canyons) as zones of enhanced species diversity. Currents and associated effects on sediments and food supply, as well as variations in surface geology and sedimentary features (i.e. habitat structure), appear to have a large influence on the distribution of mega- and macrofaunal species in these habitats. Lastly, we propose that the narrow distribution of species, the low similarity in faunal composition among sub-regions and between the deep-sea and the continental shelf, and reduced taxonomic diversity of fauna in some sub-regions, indicates that the deep-sea region bordering the Gulf of Maine harbours distinct faunal assemblages, whose persistence could be threatened by anthropogenic disturbances.

As the current state of knowledge of biodiversity in this deep-sea region is, at best, only 50% complete, a significant sampling effort will be required in future to close this gap, using multiple sampling methods in concert, to capture all (most) remaining diversity. Future additions to our knowledge will require the improvement of existing technologies (e.g. improved video sampling, automated image processing, passive acoustic detection and location), as well as the application of new technologies that go far beyond current methods (e.g. submersible technology, deep-sea instrumentation, cabled observatories). In addition, emphasis on the training of taxonomists and the support of research museums is vital to supporting future work in this, and other, deep-sea regions. These efforts will be crucial to developing a broad understanding and advancing our knowledge of biodiversity patterns in the deep-sea continental margin bordering the Gulf of Maine as we move into the post-CoML era.

## Supporting Information

Data S1 The GoMA deep-sea database. [Note: The NE Channel and Continental Rise data (380 records) provided by AM have been removed.]
(9.53 MB XLS)

Table S1 Studies cited in the GoMA deep-sea database.
(0.11 MB DOC)
