# Supplementary material for: Biodiversity of the Deep-Sea Continental Margin Bordering the Gulf of Maine (NW Atlantic): Relationships among Sub-Regions and to Shelf Systems
Source: PLoS One. 2010 Nov 19;5(11):e13832. doi: 10.1371/journal.pone.0013832 (PMC2988790; doi:10.1371/journal.pone.0013832)
Supplement: Table S1 — Studies cited in the GoMA deep-sea database. (0.11 MB DOC) [file pone.0013832.s002.doc]

Table S1. Studies cited within the GoMA deep-sea database.

| Citation found within GoMA deep-sea database | Sub-region | Reference |
| --- | --- | --- |
| Able et al. 1995 | NE Channel | [1] |
| Anon. 1974 | Canyon | [2] |
| Auster 1985 | Canyon, Continental Slope | [3] |
| Benz and Braswell 1998 | Canyon | [4] |
| Breeze et al. 1997 | NE Channel, Shelf Edge | [5] |
| Buhl-Mortensen and Mortensen 2005 | NE Channel, Shelf Edge | [6] |
| Cadrin 1995 | Canyon | [7] |
| Campbell et al. 1984 | Canyon | [8] |
| Colton 1972 | NE Channel | [9] |
| Cooper 1977 | Canyon | [10] |
| Cooper, Shephard, et al. 1987 | Canyon | [11] |
| Cooper, Valentine, et al. 1987 | Canyon | [12] |
| Corliss and Emerson 1990 | Continental Slope | [13] |
| Corliss 1991 | Continental Slope | [14] |
| Evseenko 1982 | Shelf Edge | [15] |
| Fisheries Resource Conservation Council 1998 | NE Channel | [16] |
| Frank and Widder 2002 | Canyon | [17] |
| Gass and Willison 2005 | NE Channel | [18] |
| Greene et al. 1988 | Canyon | [19] |
| Harding et al. 1997 | Canyon | [20] |
| Hartel etal 2008 | Continental Slope, Continental Rise, Seamount | [21] |
| Hecker et al. 1980 | Canyon | [22] |
| Hecker et al. 1983 | Canyon | [23] |
| Johnsen and Widder 2001 | Canyon | [24] |
| Koeller 2000 | NE Channel | [25] |
| Langton & Uzmann 1988 | NE Channel | [26] |
| Link 2004 | NE Channel | [27] |
| Maciolek etal 1985 | Canyon | [28] |
| Maciolek etal 1987 | Canyon | [29] |
| MacIsaac et al. 2000 | NE Channel | [30] |
| McClelland et al. 1985 | NE Channel | [31] |
| Mahon 1997 | NE Channel | [32] |
| Mahon et al. 1984 | NE Channel | [33] |
| Markle and Scott 1979 | NE Channel | [34] |
| Martini et al. 1998 | NE Channel | [35] |
| McElman and Elner 1982 | Continental Slope, NE Channel | [36] |
| McGlade and Boulding 1986 | NE Channel | [37] |
| McKenzie 1966 | Continental Slope | [38] |
| Metaxas and Davis 2005 | NE Channel | [39] |
| Metaxas and Giffin 2004 | NE Channel | [40] |
| Moore etal 2001 | Seamount | [41] |
| Moore etal 2003 | Seamount | [42] |
| Moore et al. 2003b | Seamount | [43] |
| Moore etal 2004 | Seamount | [44] |
| Moore et al. 2008 | Seamount | [45] |
| Morse et al. 1987 | NE Channel | [46] |
| Mortensen and Buhl-Mortensen 2004 | NE Channel | [47] |
| Musick 1974 | NE Channel | [48] |
| Neff et al. 1989 | Canyon | [49] |
| Nizinski 2003 | Canyon | [50] |
| Scott 1981 | NE Channel | [51] |
| Scott 1983 | NE Channel | [52] |
| Scott 1987 | NE Channel | [53] |
| Scott 1988 | NE Channel | [54] |
| Shepard etal 1986 | Canyon | [55] |
| Skud 1969 | Canyon | [56] |
| Stasko 1978 | Canyon | [57] |
| Stasko and Gordon 1983 | NE Channel | [58] |
| Stasko and Pye 1980 | Canyon | [59] |
| Stone and Bailey 1978 | Canyon | [60] |
| Stone and Bailey 1980 | Canyon, Continental Slope | [61] |
| Strong and Hanke 1995 | NE Channel | [62] |
| Theroux and Wigley 1998 | Continental Slope, Seamount, Shelf Edge | [63] |
| Tremblay and Sinclair 1986 | NE Channel | [64] |
| Uzmann et al. 1977 | Canyon | [65] |
| Valentine etal 1980 | Canyon | [66] |
| Waring 1994 | NE Channel | [67] |
| Watanabe et al. 2009 | NE Channel | [68] |
| Watling 1998 | NE Channel | [69] |
| Watling and Auster 2005 | Canyon, Continental Slope, Continental Rise, NE Channel, Shelf Edge | [70] |
| Wildish et al. 1992 | NE Channel | [71] |
| Williams 1988 | Canyon, Continental Slope | [72] |
| Youngbluth et al. 1989 | Canyon | [73] |

References

[1].     Able KW, Fahay MP, Shepherd GR. (1995) Early life history of black sea bass, *Centropristis striata*, in the mid-Atlantic Bight and a New Jersey estuary. Fish Bull 93: 429-445.

[2].     Anon. (1974) Deepwater survey of underused red crabs. Fishing News International 13(12): 38-41.

[3].     Auster PJ. (1985) Some observations of fish orientation to current direction and effects on predator-prey interactions. NAFO Sci Coun Studies 8: 53-55.

[4].     Benz GW, Braswell JS. (1998) Morphology of the Deep-Sea Copepod *Bobkabata kabatabobbus* (Lernaeosoleidae: Poecilostomatoida) and Amended Diagnosis of Lernaeosoleidae. The Journal of Parasitology 84(1): 109-113.

[5].     Breeze H, Davis DS, Butler M, Kostylev V. (1997) Distribution and status of deep-sea corals off Nova Scotia. Marine Issues Committee Special Publication Number 1. Ecology Action Center 58 p.

[6].     Buhl-Mortensen L, Mortensen PB. (2005) Distribution and diversity of species associated with deep-sea gorgonian corals off Atlantic Canada. In: Freiwald A, Roberts JM, editors. Cold-water Corals and Ecosystems. Berlin Heidelberg: Springer-Verlag. pp. 849-879.

[7].     Cadrin SX. (1995) Discrimination of American lobster (*Homarus americanus*) stocks off southern New England on the basis of secondary sex character allometry. Can J Fish Aquat Sci 52: 2712-2723.

[8].     Campbell A, Graham DE, MacNichol HJ, Williamson AM. (1984) Movements of tagged lobsters released on the continental shelf from Georges Bank to Baccaro Bank, 1971-73. Can Tech Rep Fish Aquat Sci1288.

[9].     Colton Jr. JB. (1972) Temperature trends and the distribution of groundfish in continental shelf waters, Nova Scotia to Long Island. Fish Bull 70(3): 637-657.

[10].     Cooper RA. (1977) Migration and dispersion of tagged American lobsters (*Homarus americanus*) on the New England continental shelf. B. F. Phillips and J. S. Cobb. Workshop on lobster and rock lobster ecology and physiology. 30 p.

[11].     Cooper RA, Shepard A, Valentine P, Uzmann J, R., Hulbert A. (1987) Pre and post drilling benchmarks and monitoring data of ocean floor fauna, habitats, and contaminant loads on Georges Bank and its submarine canyons. NOAA Symp Ser for Undersea Res 2(2): 17-48.

[12].     Cooper RA, Valentine P, Uzmann JR, Slater RA. (1987) Submarine canyons. In: Backus R, editor. Georges Bank. Massachusetts Institute of Technology Press.

[13].     Corliss BH, Emerson S. (1990) Distribution of Rose Bengal stained deep-sea benthic foraminifera from the Nova Scotian continental margin and Gulf of Maine. Deep-Sea Res 37(3): 381-400.

[14].     Corliss BH. (1991) Morphology and microhabitat preferences of benthic foraminifera from the northwest Atlantic Ocean. Mar Micropaleontol 17: 195-236.

[15].     Evseenko SA. (1982) Ichthyoplankton of slope and Gulf Stream waters off Nova Scotia in late autumn 1974. J Northw Atl Fish Sci 3: 127-139.

[16].     Fisheries Resource Conservation Council. (1998) 1999 Conservation requirements for Scotian Shelf and Bay of Fundy groundfish stocks, redfish stocks, Units 1-3 and 3-0 and groundfish stocks in Division 3Ps. Rep Fish Resour Conserv Counc 98(6): 67.

[17].     Frank TM, Widder EA. (2002) Effects of a decrease in downwelling irradiance on the daytime vertical distribution patterns of zooplankton and micronekton. Mar Biol 140: 1181–1193.

[18].     Gass SE, Willison JHM. (2005)
An assessment of the distribution of deep-sea corals in Atlantic Canada by using both scientific and local forms of knowledge. In: Freiwald A, Roberts JM, editors. Cold-water Corals and Ecosystems. Berlin Heidelberg: Springer-Verlag. pp. 223-245.

[19].     Greene CH, Wiebe PH, Burczynski J, Youngbluth MJ. (1988) Acoustical Detection of High-Density Krill Demersal Layers in the Submarine Canyons off Georges Bank. Science 241(4863): 359-361.

[20].     Harding GC, Kenchington EL, Bird CJ, Pezzack DS, Landry DC. (1997) Genetic relationships among subpopulations of the American lobster (Homarus americanus) as revealed by random amplified polymorphic DNA. Can J Fish Aquat Sci 54(8): 1762-1771.

[21].     Hartel KE, Kenaley CP, Galbraith JK, Sutton TT. (2008) Additional Records of Deep-sea Fishes from off Greater New England. Northeast Nat 15(3): 317-334.

[22].     Hecker B, Blechschmidt G, Gibson P. (1980) Epifaunal zonation and community structure in three Mid- and North Atlantic canyons. Final report for the Bureau of Land Management. United States Department of the Interior. 139 p.

[23].     Hecker B, Logan DT, Gandarillas FE, Gibson PR. (1983) Megafaunal assemblages in Lydonia Canyon, Baltimore Canyon, and selected slope areas. In: Anonymous Canyon and Slope Processes Study. Volume III – Biological Processes. : Final Report prepared for the U .S. Department of the Interior, Minerals Management Service, Washington, D .C. under Contract No . 14-12-001-29178. pp. 1-140.

[24].     Johnsen S, Widder EA. (2001) Ultraviolet absorption in transparent zooplankton and its implications for depth distribution and visual predation. Mar Biol 138: 717-730.

[25].     Koeller P. (2000) Relative importance of abiotic and biotic factors to the management of the northern shrimp (*Pandalus borealis*) fishery on the Scotian Shelf. J Northw Atl Fish Sci 27: 21-33.

[26].     Langton R, Uzmann J. (1988) A survey of the macrobenthos in the Gulf of Maine using manned submersibles. Benthic Productivity and Marine Resources of the Gulf of Maine. National Undersea Research Program Research Report No. 88-3. 131-138 p.

[27].     Link JS. (2004) Using fish stomachs as samplers of the benthos: integrating long-term and broad scales. Mar Ecol Prog Ser 269: 265-275.

[28].     Maciolek-Blake NJ, Grassle JF, Blake JA, Neff JM. (1985) Georges Bank Infauna Monitoring Program: Final report for the third year of sampling. Final Report to US Dept of Interior, Minerals Management Service, Washington, DC. Battelle New England Marine Research Laboratory, Duxbury, Massachusetts.

[29].     Maciolek N, Grassle JF, Hecker B, Brown B, Blake JA, et al. (1987) Study of biological processes on the U.S. North Atlantic slope and rise. Final report prepared for U.S. Department of the Interior, Minerals Management Service, Washington, D.C. under Contract No. 14−12−0001−30064 NTIS No. PB 88−196514/AS. 364 pp + Appendices A–L .

[30].     MacIsaac K, Bourbonnais C, Kenchington E, Gordon Jr. DG, Gass S. (2001) Observations on the occurrence and habitat preference of corals in Atlantic Canada. In: Willison JHM, editor. Proceedings of the First International Symposium on Deep-Sea Corals. Halifax, NS, Canada: Ecology Action Centre. pp. 58-75.

[31].     McClelland G, Misra RK, Martell DJ. (1985) Variations in Abundance of Larval Anisakines, Sealworm (*Pseudoterranova decipiens*) and Related Species, in Eastern Canadian Cod and Flatfish. Can Tech Rep Fish Aquat SciNo. 1392: 70 p.

[32].     Mahon R. (1997) Demersal fish assemblages from the Scotian Shelf and Bay of Fundy, based on trawl survey data (1970-1993). Canadian Manuscript Report of Fisheries and Aquatic Sciences No. 2426: 43 p.

[33].     Mahon R, Smith RW, Bernstein BB, Scott JS. (1984) Spatial and temporal patterns of groundfish distribution on the Scotian Shelf and in the Bay of Fundy, 1970-1981. Can Tech Rep Fish Aquat SciNo. 1300.

[34].     Markle DF, Scott WB. (1979) New and rare records of Canadian fishes and the influence of hydrography on resident and nonresident Scotian Shelf ichthyofauna. Can J Fish Aquat Sci 37: 49-65.

[35].     Martini F, Lesser MP, Heiser JB. (1998) A population profile for hagfish, *Myxine glutinosa*, in the Gulf of Maine. Part 2: Morphological variation in populations of *Myxine* in the North Atlantic Ocean. Fish Bull 96: 516-524.

[36].     McElman JF, Elner RW. (1982) Red crab (*Geryon quinquedens*) trap survey along the edge of the Scotian Shelf, September 1980. Can Tech Rep Fish Aquat SciNo. 1084.

[37].     McGlade JM, Boulding EG. (1986) The Truss: a geometric and statistical approach to the analysis of form in fishes. Can Tech Rep Fish Aquat SciNo. 1457.

[38].     McKenzie RA. (1966) Canadian Atlantic offshore lobster and red crab investigations, 1966. Fisheries Research Board of Canada 895: 38 p.

[39].     Metaxas A, Davis JE. (2005) Megafauna associated with assemblages of deep-water corals on the Scotian Slope. Journal of the Marine Biological Association of the UK 85: 1381-1390.

[40].     Metaxas A, Giffin B. (2004) Dense beds of the ophiuroid, *Ophiacantha abyssicola*, on the continental slope off Nova Scotia, Canada. Deep-Sea Research I 51: 1307-1317.

[41].     Moore JA, Vecchione M, Hartel KE, Collette BB, Galbraith JK, et al. (2001) Biodiversity of Bear Seamount, New England Seamount Chain: Results of Exploratory Trawling. Scientific Council Meeting September 2001 Serial No. N4549. Northwest Atlantic Fisheries Organization No. NAFO SCR Doc. 01/155. .

[42].     Moore JA, Vecchione M, Collette BB, Gibbons R, Hartel KE, et al. (2003) Biodiversity of Bear Seamount, New England Seamount chain: results of exploratory trawling. Journal of Northwest Atlantic Fisheries Science 31: 363-372.

[43].     Moore JA, Hartel KE, Craddock JE, Galbraith JK. (2003) An annotated checklist of deepwater fishes from off New England, with 110 range extensions and 81 new records off New England. Northeast Nat 10(2): 159-248.

[44].     Moore JA, Vecchione M, Collette BB, Gibbons R, Hartel KE. (2004) Selected fauna of Bear Seamount (New England Seamount chain), and the presence of “natural invader” species. Arch Fish Mar Res 51(1-3): 241–250.

[45].     Moore J, Auster P, Calini D, Heinonen K, Barber K, et al. (2008) The false boarfish *Neocyttus helgae* in the western North Atlantic. Bulletin of the Peabody Museum of Natural History 49: 31-41.

[46].     Morse WW, Fahay MP, Smith WG. (1987) MARMAP surveys of the continental shelf from Cape Hatteras, North Carolina, to Cape Sable, Nova Scotia (1977-1984). Atlas No. 2. Annual distribution patterns. NOAA Technical Memorandum. No. NMFS-F/NEC-47. .

[47].     Buhl-Mortensen L, Mortensen PB. (2004) Crustaceans associated with the deep-water gorgonian corals *Paragorgia arborea* (L., 1758) and *Primnoa resedaeformis* (Gunn.,1763). Journal of Natural History 38(10): 1233–1247.

[48].     Musick JA. (1974) Seasonal distribution of sibling hakes, *Urophycis chuss* and *U. tenuis* (Pisces, Gadidae) in New England. Fish Bull 72(2): 481-495.

[49].     Neff JM, Bothner MH, Maciolek NJ, Grassle JF. (1989) Impacts of Exploratory Drilling for Oil and Gas on the Benthic Environment of Georges Bank. Marine Environmental Research 27: 77-114.

[50].     Nizinski MS. (2003) Annotated checklist of decapod crustaceans of Atlantic coastal and continental shelf waters of the United States. Proc Biol Soc Wash 116(1): 96-157.

[51].     Scott JS. (1981) Summer distribution of groundfishes on the Scotian shelf. Fish. Mar. Serv. Tech. Rep (Bottom Trawl Surveys). pp. 181-193.

[52].     Scott JS. (1983) Inferred spawning area and seasons of groundfishes on the Scotian Shelf. Can Tech Rep Fish Aquat Sci No. 1219.

[53].     Scott JS. (1987) Matrices of co-occurrences of fish species on the Scotian Shelf and in the Bay of Fundy. Can Tech Rep Fish Aquat Sci No. 1581. 57 p.

[54].     Scott JS. (1988) Seasonal spatial distributions of groundfishes of the Scotian Shelf and Bay of Fundy , 1974-79 and 1908-84. Can Tech Rep Fish Aquat Sci No. 1653. 8 p.

[55].     Shepard AN, Theroux RB, Cooper RA, Uzmann JR. (1986) Ecology of Ceriantharia (Coelenterata, Anthozoa) of the Northwest Atlantic from Cape Hatteras to Nova Scotia. Fish Bull 84(3): 625-646.

[56].     Skud BE. (1969) The effect of fishing on size composition and sex ratio of offshore lobster stocks. FiskDir Skr Ser HavUnders 15: 295-309.

[57].     Stasko A. (1978) Inshore-offshore SW Nova Scotia lobster stock interaction: a hypothesis. Canadian Atlantic Fisheries Scientific Advisory Committee. No. CAFSAC Res. Doc. 78/37.

[58].     Stasko AB, Gordon DJ. (1983) Distribution and relative abundance of lobster larvae off southwestern Nova Scotia, 1977-1978. Can Tech Rep Fish Aquat SciNo. 1175.

[59].     Stasko AB, Pye RW. (1980) Canadian offshore lobster fishery trends. CAFSAC Res. Doc. 80/56.

[60].     Stone H, Bailey R. (1978) Preliminary assessment of the red crab resource off the Scotian Shelf. Canadian Atlantic Fisheries Scientific Adivsory Committee Res. Doc. 78/38.

[61].     Stone H, Bailey RFJ. (1980) A survey of the red crab resource on the continental slope, NE Georges Bank and western Scotian Shelf. Can Tech Rep Fish Aquat SciNo. 977. 9 p.

[62].     Strong M, Hanke A. (1995) Diversity of finfish species in the Scotia-Fundy region. Can Tech Rep Fish Aquat SciNo. 2017.

[63].     Theroux RB, Wigley RL. (1998) Quantitative composition and distribution of the macrobenthic invertebrate fauna of the continental shelf ecosystems of the Northeastern United States. NOAA Technical Report. U.S. Department of Commerce No. NOAA Technical Report NMFS 140 p.

[64].     Tremblay MJ, Sinclair MM. (1986) The horizontal distribution of larval sea scallops (*Placopecten magellanicus*) in the Bay of Fundy, on the Scotian Shelf and on Georges Bank. No. NAFO Scientific Council Research Document 86/98.

[65].     Uzmann J, R., Cooper R, A., Theroux R, B., Wigley R, L. (1977) Synoptic comparison of three sampling techniques for estimating abundance and distribution of selected megafauna: submersible VS camera sled VS otter trawl. Marine Fisheries Review 39: 12.

[66].     Valentine PC, Uzmann JR, Cooper RA. (1980) Geology and biology of Oceanographer submarine canyon. Mar Geol 38: 283-312.

[67].     Waring GT. (1994) Spatial and temporal patterns in harbour seal entanglements in the Gulf of Maine sink gillnet fishery. ICES Council Meeting Papers 1994/P:4.

[68].     Watanabe S, Metaxas A, Sameoto JA, Lawton P. (2009) Patterns in abundance and size of two deep-water gorgonian octocorals, in relation to depth and substrate features off Nova Scotia. Deep-Sea Res 56: 2235-2248.

[69].     Watling L. (1988) Benthic fauna of soft substrates in the Gulf of Maine. In: Dorsey EM, Pederson J, editors. Effects of fishing gear on the sea floor of New England. Boston, MA (USA): Conservation Law Foundation. pp. 20-30.

[70].     Watling L, Auster PJ. (2005) Distribution of deepwater alcyonacea off the northeast coast of the United States. In: Freiwald A, Roberts JM, editors. Cold-water Corals and Ecosystems. Berlin Heidelberg: Springer-Verlag. pp. 279-296.

[71].     Wildish DJ, Wilson AJ, Frost B. Benthic boundary layer macrofauna of Browns Banks, northwest Atlantic, as potential prey of juvenile benthic fish. Can J Fish Aquat Sci 49: 91-98.

[72].     Williams A, B. (1988) Notes on decapod and euphausiid crustaceans, continental margin, western Atlantic, Georges Bank to western Florida, USA. Fishery Bulletin 85(1): 67-76.

[73].     Youngbluth MJ, Bailey TG, Davoll PJ, Jacoby CA, Blades-Eckelbarger PI, et al. (1989) Fecal pellet production and diel migratory behavior by the euphausiid *Meganyctiphanes norvegica* effect benthic-pelagic coupling. Deep-Sea Res 36(10): 1491-1501.
